# The catabolism of 3,3’-thiodipropionic acid in *Variovorax paradoxus* strain TBEA6: A proteomic analysis

**DOI:** 10.1371/journal.pone.0211876

**Published:** 2019-02-11

**Authors:** Viktoria Heine, Christina Meinert-Berning, Janina Lück, Nadine Mikowsky, Birgit Voigt, Katharina Riedel, Alexander Steinbüchel

**Affiliations:** 1 Institut für Molekulare Mikrobiologie und Biotechnologie, Westfälische Wilhelms-Universität, Münster, Germany; 2 Institut für Mikrobiologie, Ernst-Moritz-Arndt-Universität, Greifswald, Germany; 3 Environmental Science Department, King Abdulaziz University, Jeddah, Saudi Arabia; Leibniz-Institut fur Naturstoff-Forschung und Infektionsbiologie eV Hans-Knoll-Institut, GERMANY

## Abstract

*Variovorax paradoxus* strain TBEA6 is one of the few organisms known to utilize 3,3’-thiodipropionate (TDP) as the only source of carbon and energy. It cleaves TDP to 3-mercaptopropionate (3MP), which is a direct precursor for polythioester synthesis. To establish this process in *V*. *paradoxus* TBEA6, it is crucial to unravel its TDP metabolism. Therefore, a proteomic approach with subsequent deletion of interesting genes in the bacterium was chosen. Cells were cultivated with D-gluconate, TDP or 3-sulfinopropionate as the only carbon sources. Proteins with high abundances in gels of cells cultivated with either of the organic sulfur compounds were analyzed further. Thereby, we did not only confirm parts of the already postulated TDP metabolism, but also eight new protein candidates for TDP degradation were detected. Deletions of the corresponding genes (two enoyl-CoA hydratases (Ech-20 and Ech-30), an FK506-binding protein, a putative acetolactate synthase, a carnitinyl-CoA dehydratase, and a putative crotonase family protein) were obtained. Only the deletions of both Ech-20 and Ech-30 led to a TDP negative phenotype. The deletion mutant of VPARA_05510, which encodes the putative crotonase family protein showed reduced growth with TDP. The three genes are located in one cluster with genes proven to be involved in TDP metabolism. Thermal shift assays showed an increased stability of Ech-20 with TDP-CoA but not with TDP. These results indicate that Ech-20 uses TDP-CoA as a substrate instead of TDP. Hence, we postulate a new putative pathway for TDP metabolism. Ech-30 interacts with neither TDP-CoA nor TDP but might interact with other CoA-activated intermediates of the proposed pathway. Further enzyme characterization is necessary to unravel the complete pathway from TDP to 3MP.

## Introduction

The species *Variovorax paradoxus* belongs to the β-proteobacteria and occurs in soil and water as well as on plants [1]. Different strains of this species utilize a variety of metabolic pathways as its name indicates. Not only are simple carbon sources such as succinate or gluconate possible nutrients, but xenobiotics and recalcitrant chemicals such as aromatic sulfonates, polychlorinated biphenyls, chitin or linuron can also be metabolized by different strains of this species. Furthermore, environmental pollutants such as arsenite, heavy metal ions, and halogenated, aliphatic or cyclic hydrocarbons are tolerated by some strains. Comparative genomics of different *V*. *paradoxus* strains (B4, EPS, S110 and TBEA6) showed that *V*. *paradoxus* strain TBEA6 possesses four unique gene clusters. These clusters may exclusively enable the strain to use 3,3’-thiodipropionate (TDP) as the only carbon source [1, 2]. The intermediate resulting from TDP cleavage, 3-mercaptopropionate (3MP), is a possible precursor substrate for the biosynthesis of the bioplastic poly(3MP) [3].

Bioplastic is an important topic in today’s time [4, 5]. Petrol-based plastic is non-degradable and its production diminishes the stocks of fossil resources. Bioplastics, e.g. polyhydroxyalkanoates (PHA), are produced from alternative substrates and are microbially degradable. In polythioesters (PTEs), thioester bonds determine the structure of the biotechnological produced persistent polymer [3]. Therefore, metabolic engineering of different bacterial strains was performed to find an optimized genetically modified PTE production strain. To establish *V*. *paradoxus* TBEA6 for the production of PTEs (e.g. to obtain an optimal supply of 3MP), characterization of the entire TDP metabolism is necessary. Metabolic engineering of strains as *Ralstonia eutropha* [6–9], *Advenella mimigardefordensis* strain DPN7^T^ [10], and *Escherichia coli* [11, 12] was successful and resulted in the production of different homo- and heteropolymers [4, 5, 10, 13]. While synthesis of poly(3MP) was already established in *A*. *mimigardefordensis* strain DPN7^T^, production with *V*. *paradoxus* TBEA6 has not been accomplished, yet. Better manageable laboratory conditions concerning *V*. *paradoxus* TBEA6 might result in a more feasible poly(3MP) production.

**Fig 1:**
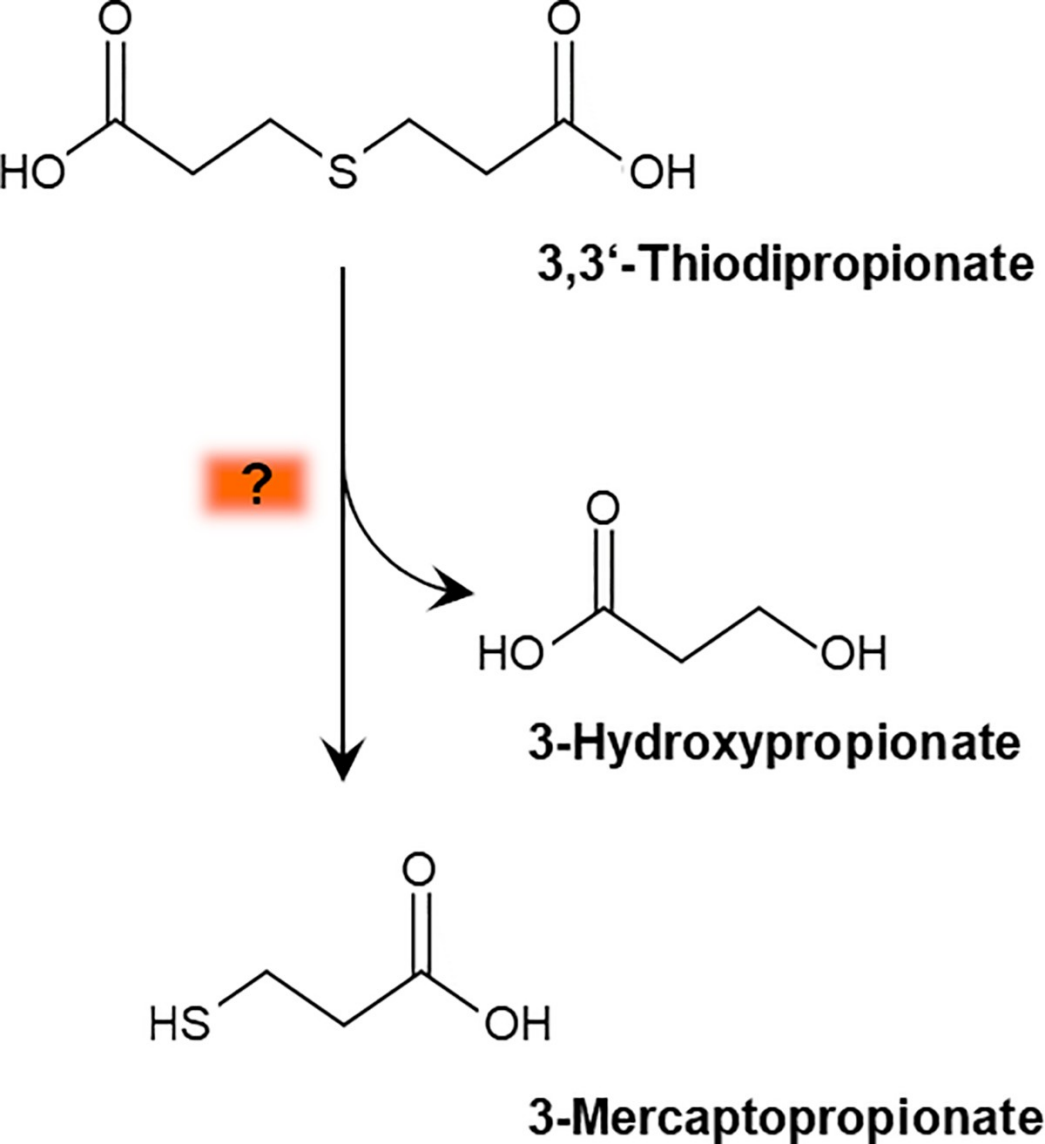
Synthesis of 3MP from TDP in *V*. *paradoxus* TBEA6. The cleavage of TDP has not been characterized until now.

Parts of the metabolism of TDP, especially the import of TDP and its initial catabolism, are still unknown. For the import of TDP into the cells, it has been hypothesized that either the tripartite tricarboxylate transport system (TTT system) or the tripartite ATP-independent periplasmatic transporter (TRAP system) convey TDP to the cytoplasm [1]. In a previous study, 3MP and traces of 3-hydroxypropionate were identified as expected intermediates of TDP degradation [2]. The detailed reaction for TDP cleavage has not been identified until now as indicated in Fig 1. Initially, a FAD-dependent oxidoreductase Fox (VPARA_05580) was assumed to catalyze this reaction. Insertions of Tn*5*::*mob* in the corresponding gene led to impaired growth of the mutants with TDP [2]. However, a recent study showed that Fox is not directly involved in TDP catabolism but has been identified as a 2-hydroxy acid specific dehydrogenase [14]. In the downstream pathway 3HP or a similar intermediate enters the central metabolism while 3MP is processed to 3-sulfinopropionate (3SP) by a cysteine dioxygenase-like 3MP-dioxygenase (Mdo) [2]. The ATP-dependent CoA-ligase SucCD [15, 16] or the succinyl-CoA-dependent CoA-transferase Act [17] activate the reaction product 3SP by addition of CoA. This intermediate is cleaved into propionyl-CoA and sulfite by the desulfinase AcdA [18, 19]. Propionyl-CoA is transferred to the central metabolism while the detoxification of sulfite most likely occurs at the cell membrane [1]. A Fe/S molybdopterin-dependent sulfite-oxidizing enzyme (SoeABC) might be responsible for the oxidation of the toxic compound as shown in *Allochromatium vinosum* [20] and *Variovorax paradoxus* B4[21, 22]. Sulfite is oxidized to sulfate and is most probably exported by a sulfite/sulfate exporter (Pse) [23, 24].

In late metabolic stages, propionyl-CoA is processed via three different possible pathways: the malonate semialdehyde pathway, the methylmalonyl-CoA pathway, and the methylcitrate cycle. The involvement of all three pathways was postulated based on *in silico* analysis by Wübbeler et al. [1]. In the malonate semialdehyde pathway, several dehydrogenases and hydratases convert propionyl-CoA via malonate semialdehyde to acetyl-CoA. The second pathway consists of a semialdehyde dehydrogenase and a methylmalonyl-CoA mutase catalyzing the reactions via methylmalonyl derivatives to succinyl-CoA. Methylcitrate and methylisocitrate are intermediates of the third pathway catalyzed by corresponding dehydratases. Succinyl-CoA and acetyl-CoA from all three pathways then enter the central metabolism.

In this study, we applied several methods such as proteomic analyses, marker-free gene deletion, growth experiments, and enzyme characterization to elucidate the initial catabolic reactions with TDP. The resulting protein profiles provided several promising proteins; two of them were annotated as putative enoyl-CoA hydratases (Ech). Deletion of both Echs resulted in impaired growth with TDP. Therefore, both genes appeared to be essential for the utilization of TDP by *V*. *paradoxus* strain TBEA6. Their function in TDP metabolism and degradation was further analyzed during this study. Additionally, a putative crotonase family protein has been hypothesized to be involved in TDP catabolism, since deletion of this gene reduced the growth of *V*. *paradoxus* TBEA6 with TDP. The study provided new insights in TDP metabolism, and we are now able to propose a potential reaction pathway from TDP to 3MP and other intermediates.

## Materials and methods

### Cultivation and cell harvest

*E*. *coli* strains were cultivated overnight at 37°C on solid or in liquid Lysogeny Broth containing ampicillin (75 μg ml^-1^) or tetracycline (12.5 μl ml^-1^) depending on the resistance of the strain. *V*. *paradoxus* was cultivated overnight at 30°C in mineral salt medium (MSM) [25] or Nutrient Broth in liquid cultures or for 3 to 5 days on solid medium (solidified with 2% Agar-Agar). MSM was completed with different carbon sources (60 mM gluconate, TDP or 3SP). Liquid cultures were incubated on a rotary shaker at 130 rpm (New Brunswick Scientific Co., Inc., NJ, USA) in Erlenmeyer flasks with baffles to guarantee optimal oxygen supply. Strains used in this study are listed in Table 1.

**Table 1:** Strains and plasmids used in this study.

Strain	Relevant geno- and phenotype	Reference
*Variovorax paradoxus* TBEA6	Utilizes TDP as sole carbon source	[2]
*Escherichia coli* TOP10	Ф80*lacZ*ΔM15, Δ*lacX*74, *deoR*, *recA*1, *araD*139, *galK*, *aa*I*U*, Δ(*ara-leu*)7697, *endA*1	Invitrogen, Darmstadt, Germany
*Escherichia coli* S17-1	*thi*-1, *proA*, *hsdR*17 (rk- mk+), *recA*1, *tra*-genes of plasmid RP4 integrated into the genome, F-, *mcrA*, Δ(*mrr*-*hsdRMS*-*mcrBC*), *rpsL*, *nupG*	[26]
*E*. *coli* BL21 (DE3) pLysS	F^−^, *ompT*, *hsdS*_*B*_*(r*_*B*_*−*, *m*_*B*_*−)*, *gal*, *dcm* (DE3)*/*pLysS (Cm^r^)	Novagen, Madison, USA
*E*. *coli* Rosetta (DE3) pLysS	F-, *ompT*, *hsdSB*, *(rB-*,*mB-) gal*, *dcm* (DE3), pLysSRARE, Cm^r^	Novagen, Madison, USA
*Variovorax paradoxus* TBEA6Δ*ech-20*	No growth with TDP	This study
*Variovorax paradoxus* TBEA6Δ*ech-30*	No growth with TDP	This study
*Variovorax paradoxus* TBEA6Δ*ech-20*Δ*ech-30*	No growth with TDP	This study
*Variovorax paradoxus* TBEA6Δ05510	Reduced growth with TDP	This study
**Plasmid**		
pJET1.2/blunt	*Bla*, *rep(pMB1)*, *eco47IR*	ThermoFisher Scientific Darmstadt, Germany
pJQ200mp18Tc	Tc^R^, suicide vector for gene deletion	[27]
pET-19b::*pct*_Re_	*E*. *coli* expression vector, (N-terminal His-tag, Amp^r^, T7 promoter)	[28]
pET23a(+)	*E*. *coli* expression vector (C-terminal His-tag, Amp^r^, T7 promoter)	Novagen, Madison, USA
pET32a	pBR322 *ori*, f1 *ori*, His6, Amp^r^, T7lac, Trx	Novagen, Madison, USA
pET23a(+)::e*ch-30*	*E*. *coli* expression vector (C-terminal His-tag, Amp^r^, T7 promoter) expressing *ech*-30 (VPARA_05530)	This study
pET32a(+)::e*ch-20*	*E*. *coli* expression vector (C-terminal His-tag, Amp^r^, T7 promoter) expressing *ech*-20 (VPARA_05520)	This study

To determine the growth phase of liquid cell cultures, the optical density (OD) was measured at defined time intervals. The OD was determined by three different methods depending on the experiment. 1) A spectral photometer (Thermo Spectronic GENESYS 20 Visible Spectrophotometer, Conquer Scientific, San Diego, USA) was used for general OD measurements (e.g. protein production, proteomic approach). For proteome analysis, liquid cultures were harvested 6 h after entering the stationary growth phase. 2) A Klett-Summerson photometer (Manostat Corporation, NY, USA) served for supernatant analyses to reduce the risk of contamination. 3) For growth experiments, a microplate reader (EPOCH2TC, BioTek Instruments, Winooski, USA) was used. The conditions resembled the conditions in flasks. The wells of microtiter plates contained 200 μl of medium and cells of the different strains. Each growth experiment was performed in triplicates of each culture and with controls, containing no cells. Cells were harvested by centrifugation at 4°C and 7,690 x *g* for 10 min to 30 min (Universal 320R, Hettich Lab Technology, Tuttlingen, Germany).

### Preparation of protein samples for 2D-PAGE

To obtain the proteins, cell pellets were resuspended in the equivalent volume of Tris/HCl buffer (50 mM Tris, pH 7.4) containing protease inhibitor and DNase. Afterward, the cells were disrupted at 4°C and 1000 MPa by five passages through a French press (French Pressure Cell Press KIN004, Fa. Amicon, Silver Spring, Maryland, USA). The cell debris was removed by centrifugation (7,690 x *g*, 4°C, 30 min); the supernatants were retained. Precipitation was done with acetone containing 20% (wt/vol) trichloroacetic acid and 20 mM DTT for 1 h at -20°C. After centrifugation, the supernatant was discarded. Pellets were washed with 20 ml acetone containing 20 mM DTT until the yellow pigment naturally formed by *V*. *paradoxus* TBEA6 was removed from the pellet. The pellets were dried and stored at -20°C.

### 1^st^ dimension: Isoelectric focusing for 2D-PAGE

Protein pellets were dissolved in 500 to 1000 μl rehydration buffer A (7 M urea, 2 M thiourea, 4% (wt/vol) 3-[(3-cholamidopropyl)dimethylammonio]-1-propanesulfonate, 100 mM DTT) and incubated overnight at room temperature to allow optimal rehydration. The protein solutions were centrifuged for 5 min at 14,000 x *g*; the supernatants were retained. The volume corresponding to 1.5 mg protein was complemented to 200 μl with rehydration buffer A, and 150 μl of rehydration buffer B (rehydration buffer A with 0.05% (vol/vol) Bio-Lyte 3/10 Ampholyte (Biorad, Hercules, USA), 0.05% (vol/vol) Triton X-100, and a pinch of bromophenol blue) were added. Solutions and IPG strips (ReadyStrip IPG Strips, pH 5–8, 17 cm, Biorad, Hercules, USA) were transferred into a focusing tray and covered with mineral oil. The following voltages were applied for isoelectric focusing, using a PROTEAN IEF cell (Biorad, Hercules, USA): 250 V (250 Vh), 500 V (500 Vh), 1,000 V (1,000 Vh), 6,000 V (108,000 Vh), and 500 V (until further use).

### 2^nd^ dimension: SDS-PAGE for 2D-PAGE

After preparation of the IPG strips, 12.5% (wt/vol) polyacrylamide gels (200 mm x 200 mm x 1 mm) were produced as described before [22]. IPG-strips were equilibrated for 10 min in SDS-equilibration buffer (1.5 M Tris/HCl pH 8.8, 7M urea, 2% SDS, 34.5% glycerol, and a pinch of bromophenol blue) and then for another 10 min in SDS-equilibration buffer supplemented by iodoacetamide. The strips were washed in electrode buffer (25 mM Tris/HCl, 192 mM glycine, 0.1% (wt/vol) SDS), which was also used for 2D-PAGE in a DODECA Cell (PROTEAN plus DODECA Cell, Biorad, Hercules, USA), and fixed onto the gels with sealing solution (1% agarose (wt/vol) in electrode buffer and a pinch of bromophenol blue). The PAGE was carried out with 5 V per gel for one hour and afterward with 20 V per gel until the run was finished. Afterward, gels were stained overnight (0.4% Serva Blue G 250 (wt/vol), 45% methanol (vol/vol), and 9% acetic acid (vol/vol)) and destained for 10 h (33% (vol/vol) methanol and 10% (vol/vol) acetic acid). Gels were stored in 10% acetic acid (vol/vol).

### Software-based analysis of 2D-gel images

The gels were analyzed via Delta2D software (Decodon GmbH, Greifswald, Germany) according to the manufacturer’s recommended practices. Four replicates of each condition (different carbon sources: gluconate, TDP, and 3SP) were produced in two independent biological experiments. Three gels per condition were selected for further analysis via Delta2D. Gels of identical conditions were warped to correct differences between the gels. The average gel images of all three conditions were warped to create a spot mask. Intensities of the spots were calculated due to their volume percentage in relation to the total level of protein contained in the gel. After normalization of the spot volumes by the program, statistics were performed to exclude insignificant spots. Therefore, analysis of variance (ANOVA) was applied with a p-value of 0.05. Spots with a standard deviation higher than 30% were excluded from the analysis. Spots of the different conditions were compared and set into relation. The values displayed the differences in the abundance of certain spots regarding the certain conditions (carbon sources) in contrast. Spots, which exhibited a ratio higher than two in gels of cells cultivated with TDP or 3SP, were filtered and analyzed via matrix laser desorption/ionization-time-of-flight-tandem mass spectrometry (MALDI-TOF-MS/MS).

### Mass spectrometry

Selected spots were cut from the SDS-gels and stored in 10% (vol/vol) acetic acid. The samples were prepared for MALDI-TOF-MS/MS analysis as described previously [29]. The analysis was performed with a 4800 Proteomics Analyzer (AB Sciex, Framingham, MA, USA). Spectra were recorded in the reflector mode with a focus mass of 2000 Da (mass range: 900 Da to 3700 Da). Calibration of mass spectrometry data was performed as already described [30]. The proteome database of *V*. *paradoxus* strain TBEA6 was used for the identification of peaks with peak lists from MS and MS/MS, using the Mascot engine (version 2.1.0.4).

### Isolation and transfer of DNA

Depending on the experiment, DNA was isolated with different kits following the manufacturer’s instructions. Cells were harvested from liquid cultures or agar plates and treated as stipulated. Genomic DNA was isolated using the NucleoSpin Tissue Kit (Machery-Nagel GmbH Co. KG, Düren, Germany), plasmid DNA was extracted with the GeneJET Plasmid Miniprep Kit (Fermentas, St. Leon-Tor, Germany). DNA fragments were purified from reaction mixtures by peqGOLD gel extraction kit (PEQlab Biotechnology GmbH, Erlangen, Germany). To transfer DNA to *E*. *coli* strains, competent cells were prepared and transformed using the CaCl_2_ method [31]. Conjugation was performed [32] to transfer plasmids from *E*. *coli* to *V*. *paradoxus* TBEA6.

### Modification of DNA

DNA fragments were amplified via PCR in a thermocycler (peqSTAR 2x Gradient Thermocycler, Peqlab Biotechnologie GmbH, Erlangen, Germany) using *taq* (Biomix, Bioline, London, UK) or Phusion High Fidelity polymerase (Fermentas, St. Leon-Roth, Germany). Used oligonucleotides were produced by MWG-Biotech (Ebersberg, Germany) and are listed in S1 Table. Restriction endonucleases (Fermentas, St. Leon-Rot, Germany) were used for DNA digestion; T4-Ligase (Invitrogen, Karlsruhe, Germany) was used for ligation.

### DNA sequencing

DNA samples were sequenced by MWG-Biotech (Ebersberg, Germany). Characterization of the sequences was performed using the Seqman software (DNASTAR, Wisconsin, USA).

### Construction of gene deletion suicide plasmids

Up- and downstream flanking regions of the target genes were amplified using the oligonucleotides listed in S1 Table. Both flanks were ligated and amplified again with the corresponding primers. The constructs were integrated into the suicide plasmid pJQ200mp18Tc [27], using pJET1.2/blunt as a subcloning vector. Plasmids were multiplied in *E*. *coli* TOP10 and transferred to *E*. *coli* S17-1 for further use.

### Gene deletion using the *sacB* system

The suicide plasmid was transferred from *E*. *coli* S17-1 to *V*. *paradoxus* TBEA6 via spot mating [32] for heterologous recombination. Relevant deletion mutants were identified by inoculation on solid nutrient broth medium containing 15% (wt/vol) sucrose (growth), MSM containing Tc and gluconate (no growth), and MSM containing TDP (no growth). Deletion of the target gene was verified by PCR and sequencing. Correct mutants showed no PCR product with internal primers and a truncated PCR product with external primers (S2 Table). Internal primers bind inside the gene, external primers bind outside of the flanking regions.

### Supernatant analysis

The consumption of TDP was analyzed in cultures of *V*. *paradoxus* TBEA6 and Ech mutant strains. First, cell mass was generated via cultivation with MSM containing 60 mM succinate until the cultures reached the late exponential growth phase. Cells were harvested and transferred to MSM with 50 mM TDP and 10 mM succinate. After medium exchange, samples of 5 ml were withdrawn at specific time intervals (t = 0, 24, 48, 72, 144 h) and centrifuged. Control flasks without carbon source or without cells were applied to exclude contamination and detect spontaneous degradation of TDP to 3MP in the medium. The supernatants were analyzed by gas chromatography (GC; Series 6890 GC-System, Hewlett Packard GmbH, Dortmund, Germany) using a SGE BP21 capillary column with a split ratio of 1:20, a pressure of 86.5 kPa and a split temperature of 250°C (SGE, Darmstadt, Germany). Two μl of the sample were injected at a hydrogen flow of 18.6 ml/min. For sample preparation, 2 ml of the supernatant were lyophilized, solved (2 ml methanol-sulfuric acid (20:3, (vol/vol)) and 2 ml chloroform), and incubated for 3 h at 100°C. Afterwards, 2 ml of water were added and vortexed. The organic phase was extracted and measured.

### Heterologous expression of *pct*_*Re*_, *ech-20* and *ech-30*

For heterologous expression of *pct*_*Re*_, *E*. *coli* BL21 (DE3) pLysS was transformed with pET-19b::*pct*_*Re*_. The cells were cultivated in a preculture overnight in 20 ml of LB medium containing 75 μg/ml ampicillin. The main culture was inoculated with 1% (vol/vol) of the pre-culture and incubated at 30°C. Expression of *pct*_*Re*_ was started by the addition of 1 mM IPTG at an OD_600_ 0.4 as described previously by Lindenkamp et al. [28]. Purification of recombinant Pct_Re_ was performed as described in detail [28] using Ni-Sepharose columns (His SpinTrap; GE Healthcare, Munich, Germany).

Heterologous production of Ech-20 and Ech-30 was accomplished by transformation of *E*. *coli* BL21 (DE3) pLysS with pET-23a(+)::*ech-30* and pET-32a(+)::*ech-20*. The freshly transformed cells were incubated in 20 ml LB medium overnight. From this preculture, 1% (vol/vol) was used to inoculate the main culture of 50 ml or 100 ml autoinduction medium (ZYP, [33]). The cultures were incubated for approximately 18 h on a rotary shaker at 130 rpm. Subsequently, cells were harvested (7,360 x *g*, 15 min, 4°C) and disrupted by three to five passages through a FrenchPress (4°C, 1000 MPa). Cell debris was removed by centrifugation, and the recombinant Echs were purified using Ni-Sepharose columns (His SpinTrap; GE Healthcare, Munich, Germany). Following the manufacturer´s instructions we used sodium phosphate (NaP) buffer (binding buffer: 20 mM NaP, 20 mM imidazole, 500 mM NaCl; wash buffer: 20 mM NaP, 100 mM imidazole, 500 mM NaCl; elution buffer: 20 mM NaP, 500 mM imidazole, 500 mM NaCl). For protein storage and further experiments, the elution buffer was exchanged for 20 mM NaP buffer (pH 7.4) containing 100 mM NaCl using Vivaspin 500 columns (Sartorius AG, Göttingen, Germany).

### Synthesis of CoA-esters

The transfer of CoA from acetyl-CoA to TDP was accomplished using recombinant Pct_Re_ as described by Volodina et al. [34]. Accordingly, 1 mM acetyl-CoA, 2 mM TDP, and 20 μg of Pct_Re_ were mixed in 100 mM Tris/HCl buffer (pH 8,0); the final volume amounted to 1 ml. Samples were incubated for 45 min at 30°C. To stop the reaction and denature the protein, 180 μl of trichloroacetic acid were added. The denatured Pct_Re_ was removed by centrifugation, and the formed TDP-CoA was purified as described in detail by Eggers et al. [35]. First, the used Sep Pak C18 Classic column (Waters Corporation, Milford, USA) was rinsed two times with 2.5 ml of 80% (vol/vol) methanol. The columns were equilibrated twice with 2.5 ml of a 50 mM ammonium acetate solution (pH 7.4). Additionally, the ammonium acetate solution was added to the samples in a ratio of 1:10 and applied to the columns. Subsequently, the columns were washed twice with 2.5 ml of a mixture of 80% methanol and 20% ammonium acetate buffer (50 mM, pH 4.7). The CoA esters were finally eluted with 2.5 ml of 80% (vol/vol) methanol and dried in a vacuum furnace at 30°C.

### High performance liquid chromatography/ mass spectrometry (HPLC/MS)

HPLC/MS was used to analyze the synthesized TDP-CoA. Therefore, the samples were acidified by addition of 0.1% formate and analyzed in an UltiMate 3000 HPLC apparatus (Dionex GmbH, Idstein, Germany) directly connected to an LXQ Finnigan mass spectrometer (ThermoScientific, Dreieich, Germany). An Acclaim 120 C18 Reversed-Phase LC Column (4.6 x 205 mm, 5 μm, 120 Å pores; Dionex GmbH, Idstein, Germany) was used at 30°C. Ammonium acetate buffer (50 mM, pH 4.7; eluent A) and 100% methanol (eluent B) served as eluents. The flow rate was set to 0.5 ml/min. Ramping was performed as follows: equilibration was started with 80% eluent A and 20% eluent B. After injection, within a period of 20 min, eluent B was increased in a gradient to a final concentration of 85%. Within the next 10 min, the concentration of eluent B was reduced to 20% and held for a duration of another 10 min. Detection of CoA esters was done at 259 nm with a photodiode array detector. The device was tuned by direct infusion of a 1 mM CoA trilithium salt solution at a flow rate of 10 μl/min into the ion trap of the mass spectrometer to optimize the ESI-MS system for generation of protonated molecular ions of CoA derivatives. The tuning parameters were set as follows: capillary temperature, 300° C; sheat gas flow, 12 liters/h; auxiliary gas flow, 6 liters/h; and sweep gas flow, 1 liter/h. The mass range was set to *m/z* 50 to 1,000 Da when running in the scan mode. The collision energy in the MS mode was set to 30 V.

### Thermal shift assay

Thermal shift assays were used to elucidate a stabilizing effect of putative ligands on Ech-20 and Ech-30. Therefore, 2 μM of protein, 2 mM of the potential ligand, and 5 x SyproOrange (5000 x stock solution, Sigma-Aldrich, MO, USA) were mixed in a final volume of 20 μl using a Tris/HCl (100 mM, pH 8.0) buffer system. Samples were put into 48 well plates (MicroAmpOptical 48-Well reaction plate, Applied Biosystems, Foster City, CA, USA) and sealed with optical adhesive films (MicroAmp 48-well Optical Adhesive Film, PCR Compatible, Applied Biosystems, Foster City, CA, USA). A StepOne real-time PCR system (Applied Biosystems, Forster City, CA, USA) was used to record the fluorescence during the temperature range of 20°C to 90°C following the protocol from Vivoli et al. [36].

## Results and discussion

The aim of this study was the characterization of the degradation of TDP to 3MP and 3HP. A proteomic approach was performed to detect proteins, influenced by growth of *V*. *paradoxus* TBEA6 using TDP as the only carbon source. Deletion of genes coding for two Echs and a crotonase family protein led to significant phenotypes, which were confirmed by further experiments. Results from the different approaches are described in this chapter to provide thorough information.

### Proteome analysis of *V*. *paradoxus* TBEA6

**Fig 2:**
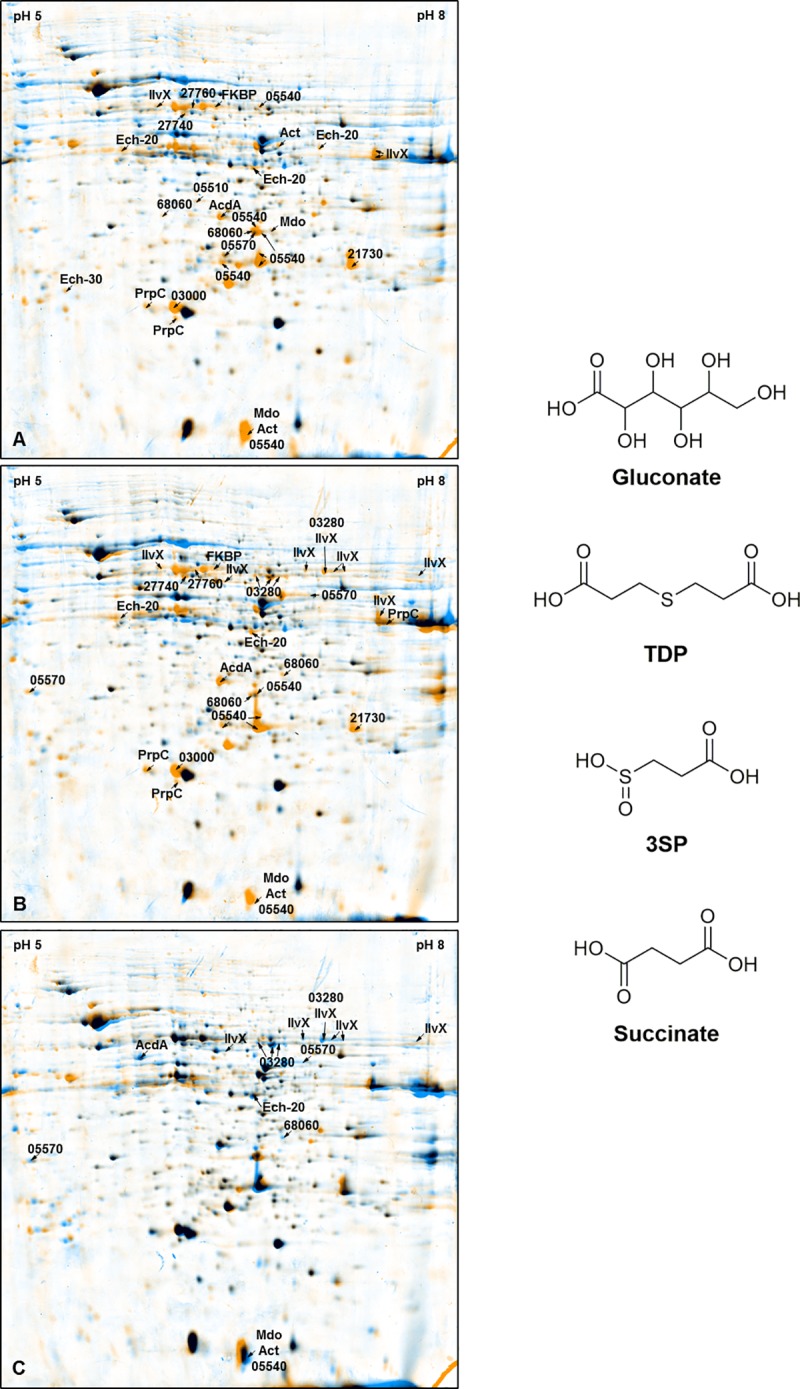
2D-gel dual views and relevant carbon sources. Left: Dual views were modeled by overlay of 2D-gels carrying the proteomic composition during cell growth with one specific carbon source. Proteins (1.5 mg per gel) were separated by isoelectric point between pH 5 and pH 8 (first dimension) and molecular weight (second dimension). Gels were stained with Coomassie Brilliant Blue and analyzed via Delta2D Software. Interesting spots were isolated and examined via MALDI-TOF. These spots are highlighted in this figure. A) TDP (orange) vs gluconate (blue); B) 3SP (orange) vs gluconate (blue); C) TDP (blue) vs 3SP (orange). Right: Gluconate, TDP, 3SP or succinate were used for several growth experiments to identify the impact of the organic sulfur compounds on the proteome.

*V*. *paradoxus* TBEA6 cells were cultivated in MSM with gluconate, TDP or 3SP (60 mM) as sole carbon source, respectively, to display differences in protein profiles. Comparisons were drawn between the 2D gels of cells cultivated with TDP and gluconate, 3SP and gluconate, and TDP and 3SP (Fig 2). Cells of all three cultures were harvested 6 h after reaching the stationary phase. The experiment was repeated to confirm the results of the first biological experiment (S1–S3 Figs; S3 and S4 Tables).

### Identification of proteins with increased abundance during cell growth with TDP and 3SP in comparison to gluconate

To characterize the initial reactions of TDP catabolism in detail, 2D gels of cells cultivated with TDP or 3SP were compared to gels of cells cultivated with gluconate. Furthermore, the comparison of the protein profiles of TDP and 3SP (Fig 2) might provide specific evidence to the process itself, because the detected proteins can be related immediately to TDP catabolism. The other comparisons relate to the complete catabolism of TDP, also including downstream steps of the postulated catabolism.

To identify proteins potentially involved in the mechanism, the protein spots were analyzed via Delta2D Software. Quantitation tables were generated, displaying the normalized spot volumes (%V) and the ratios between the spots of different conditions (S3 and S4 Tables; S1–S3 Figs). A spot mask filtered spots with ratios higher than 2 or lower than 0.5 and highlighted the most promising proteins. For our purpose, only spots with a ratio higher than 2 were considered. An increased expression of genes with TDP as a carbon source was expected to indicate genes connected to TDP catabolism. From 173 detected spots, 98 fulfilled the requirements. For 79 of these selected spots, proteins were identified and assigned to locus tags in the genome of *V*. *paradoxus* TBEA6 (Table 2).

**Table 2:** Amounts of proteins detected via 2D gel analysis for the different cultivation conditions.

Comparison	Amount of protein spots with significantly increased spot volumes (ratio ≥ 2)	Amount of spots identified with MALDI-TOF and protein databases
TDP vs gluconate	**56**	**53**
3SP vs gluconate	**58**	**51**
TDP vs 3SP	**40**	**29**

The better part of the detected proteins (59%) act in metabolic pathways, while the others catalyze processes in the biosynthesis of building blocks (28%), transport (7%), transcription (2%), DNA modification (2%), cellular stress response (1%), and protein folding (1%). The detected proteins were analyzed via *in silico* analysis and examined regarding their potential function in TDP metabolism.

### TDP metabolism

Important reactions of the TDP catabolism from 3MP and 3SP to propionyl-CoA have been described in detail [1]. The proteome analysis of this study displayed the pathway very clearly. Amongst all detected proteins, the 3-mercaptopropionate dioxygenase (Mdo) [2], the acyl-CoA dehydrogenase-like desulfinase (AcdA) [18, 19], and the succinyl-CoA-dependent CoA transferase (Act) [17] occurred with a high abundance in the 2D gels (Table 3). Regarding the metabolic part leading from propionyl-CoA to the central metabolism, we postulated three putative pathways from *in silico* analysis [1]. With the proteomic approach, we detected at least one enzyme of each postulated pathway (Table 3). For the malonate semialdehyde pathway, participation of Ech-30 and the semialdehyde dehydrogenase were observed. The semialdehyde dehydrogenase putatively performs two steps in the methylmalonyl-CoA pathway as well. The 2-methylcitrate synthase, the 2-methylcitrate dehydratase, and the methylisocitrate lyase are possibly involved in the the methylcitrate cycle. The appearance of all these enzymes supports the three postulated pathways. It is likely that they occur simultaneously. The resulting intermediates, succinyl-CoA and propionyl-CoA are common metabolic intermediates. Therefore, the three described pathways might as well be active during other metabolic reactions with alternative initial substrates.

**Table 3:** Proteins from our postulated and partly verified TDP metabolism detected via proteome analysis. The table includes the spot numbers in the 2D gels (S1–S3 Figs), the protein function and the corresponding locus tag. The highest relevant ratios of mean normalized spot volumes are indicated in black; insignificant ratios are displayed in grey.

Spot	Protein function (EC -.-.-.-)	Gene	ORF (VPARA_ XXXXX)	Ratio Vol% TDP / Vol% Gluc	Ratio Vol% 3SP / Vol% Gluc	Ratio Vol% TDP / Vol% 3SP
30, 58, 153	3MP dioxygenase Mdo	*mdo *	05600	**26.11**	**8.02**	**3.25**
53, 161	Acyl-CoA dehydrogenase-like desulfinase AcdA	* acdA*	05440	**5.58**	**3.39**	**3.63**
30, 132	Succinyl-CoA-dependent CoA transferase Act	* act*	05450	**26.11**	**8.02**	**3.25**
17	Putative enoyl-CoA hydratase (4.2.1.17) (Ech-30)	*ech*	05530	0.73	**2.21**	0.33
88, 118, 119, 120	Semialdehyde dehydrogenase		03280	**3.38**	1.32	**2.85**
8, 57, 64, 149	Semialdehyde dehydrogenase		05570	**3.65**	**6.69**	**12.95**
21, 24, 83	2-Methylcitrate synthase	* prpC*	03010	**5.89**	**5.08**	1.47
19	2-Methylcitrate dehydratase		03000	**18.44**	**20.16**	0.91
7, 60, 172	Methylisocitrate lyase		68060	**3.16**	**2.75**	**4.57**

### Proteins selected for gene deletion

Nine different proteins aroused attention due to their putative function and their high abundance in 2D gels of cells grown with TDP or 3SP in comparison to gluconate as the only carbon source (Table 4).

**Table 4:** Proteins identified via proteome analysis exhibiting an increased spot volume during cultivation of *V*. *paradoxus* TBEA6 with 3,3´-thiodipropionic acid (TDP) or 3-sulfinopropionic acid (3SP) in comparison to gluconate (Gluc). The table includes the spot numbers in the 2D gels (S1–S3 Figs), the protein function and the locus tag. The highest relevant ratios of mean normalized spot volumes are indicated in black; insignificant ratios are displayed in grey.

Spot number	Protein function (EC -.-.-.-)	Gene	ORF (VPARA_ XXXXX)	Ratio Vol% TDP / Vol% Gluc	Ratio Vol% 3SP / Vol% Gluc	Ratio Vol% TDP / Vol% 3SP
79	Putative 3-hydroxyisobutyryl-CoA hydrolase		05510	0.83	**2.56**	0.32
47, 84, 138	Putative enoyl-CoA hydratase 1 (4.2.1.17) Ech-20	*ech*	05520	**4.56**	**4.60**	**2.18**
17	Putative enoyl-CoA hydratase 1 Ech-30	*ech*	05530	0.73	**2.21**	0.33
30, 56, 59, 63, 65, 66, 114	Carnitinyl-CoA dehydratase (4.2.1.149)	*caiD*	05540	**26.11**	**20.69**	**3.25**
82, 85–88, 106, 123, 163, 164	Putative acetolactate synthase large subunit IlvX (2.2.1.6)	*ilvX*	05550	**3.86**	**6.78**	**9.56**
12	Putative FAD-linked oxidoreductase (1.-.-.-)	* *	21730	**4.12**	**4.79**	0.86
112	FK506-binding protein (5.2.1.8)	*fbp*	24900	**5.74**	**5.36**	1.07
121	Putative FAD-linked oxidoreductase (1.-.-.-)		27740	**4.53**	**3.22**	1.41
113	Putative metallo-hydrolase (3.-.-.-)		27760	**3.61**	**3.86**	0.93

**Fig 3:**
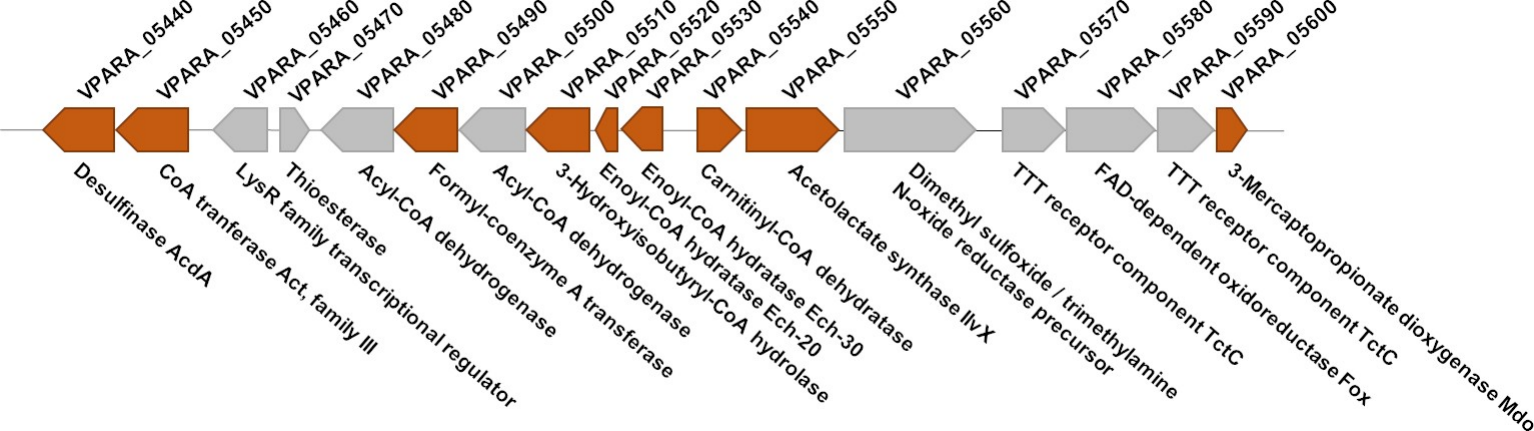
Gene cluster relevant for TDP degradation as indicated by proteome analysis, comprising *mdo*, *act* and *acdA*. Proteins with high abundance during cultivation with TDP identified by proteome analysis are marked in orange.

A putative crotonase family protein and two putative enoyl-CoA hydratases (Ech-20 and Ech-30) showed high abundance in 2D gels from cultures with both organic sulfur compounds in contrast to the control (gluconate). Also a carnitinyl-CoA dehydratase, a putative acetolactate synthase IlvX, and two putative FAD-linked oxidoreductases were analyzed due to their overrepresentation in the proteome of cells grown with TDP and 3SP. An FK506-binding protein FKBP and a putative metallo hydrolase exhibited an increased spot volume in TDP gels. Deletion was accomplished for each corresponding gene, except for VPARA_27760. It is likely that this enzyme is essential for the survival of the cell. The gene encoding the putative crotonase family protein (VPARA_05510) was deleted due to its location in the cluster comprising genes responsible for TDP catabolism (Fig 3).

Only the deletion of *ech-20*, *ech-30*, and the gene encoding the crotonase family protein resulted in an altered phenotype with TDP as the only carbon source. Deletion mutants lacking VPARA_05540, VPARA_05550, VPARA_21730, VPARA_24900, or VPARA_27740 showed no change in growth with TDP (not shown). Still, they might be involved in the general sulfur metabolism (e. g. amino acid biosynthesis, detoxification) due the high abundance of the respective proteins. Some of these proteins were detected in the same spots as proteins crucial for TDP metabolism. High abundance of the carnitinyl-CoA dehydratase (VPARA_05540) and the acetlocatate synthase (VPARA_05550) might be related to their genetic vicinity (Fig 3) to important genes of the TDP metabolism. For the other genes (VPARA_21730, VPARA_27740, VPARA_27760), no connections to the TDP catabolism were identified.

### Enoyl-CoA hydratases (VPARA_05520, VPARA_05530) and a crotonase family protein (VPARA_05510)

**Fig 4:**
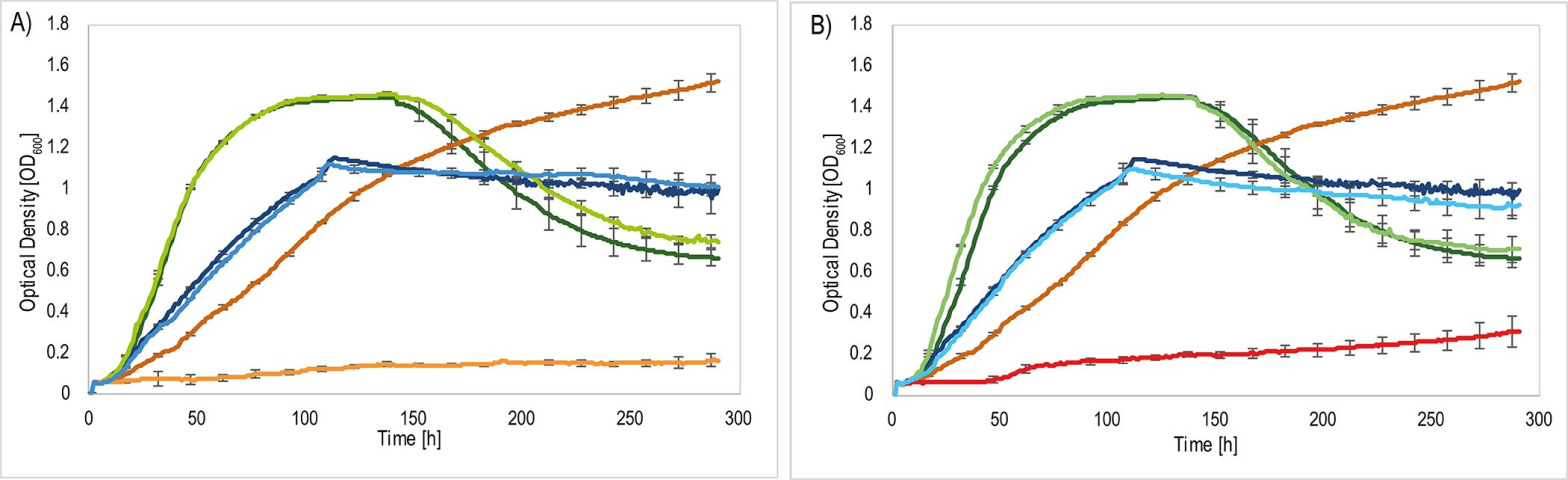
Growth curves of the wildtype *V*. *paradoxus* TBEA6 wildtype in comparison to the mutants Δ*ech-20* and Δ*ech-30*. Cells were cultivated in MSM containing 60 mM gluconate, TDP or 3SP. *V*. *paradoxus* TBEA6 with gluconate (dark green), *V*. *paradoxus* TBEA6 with TDP (orange), *V*. *paradoxus* TBEA6 with 3SP (dark blue) (in A and B), A) *V*. *paradoxus* TBEA6 Δ*ech-20* with gluconate (light green), *V*. *paradoxus* TBEA6 Δ*ech-20* with TDP (yellow), *V*. *paradoxus* TBEA6 Δ*ech-20* with 3SP (light blue). B) *V*. *paradoxus* TBEA6 Δ*ech-30* with gluconate (light green), *V*. *paradoxus* TBEA6 Δ*ech-30* with TDP (red), *V*. *paradoxus* TBEA6 Δ*ech-30* with 3SP (light blue). The experiment was performed in triplicate. Standard deviations are indicated as error bars.

Ech-20 and Ech-30 were of special interest, as they seemed to be involved in TDP metabolism. Deletion mutants lacking one or both *ech* genes were unable to grow with TDP. Growth with 3SP and gluconate was unaffected. The observed phenotype was confirmed in liquid cultures (Fig 4).

**Fig 5:**
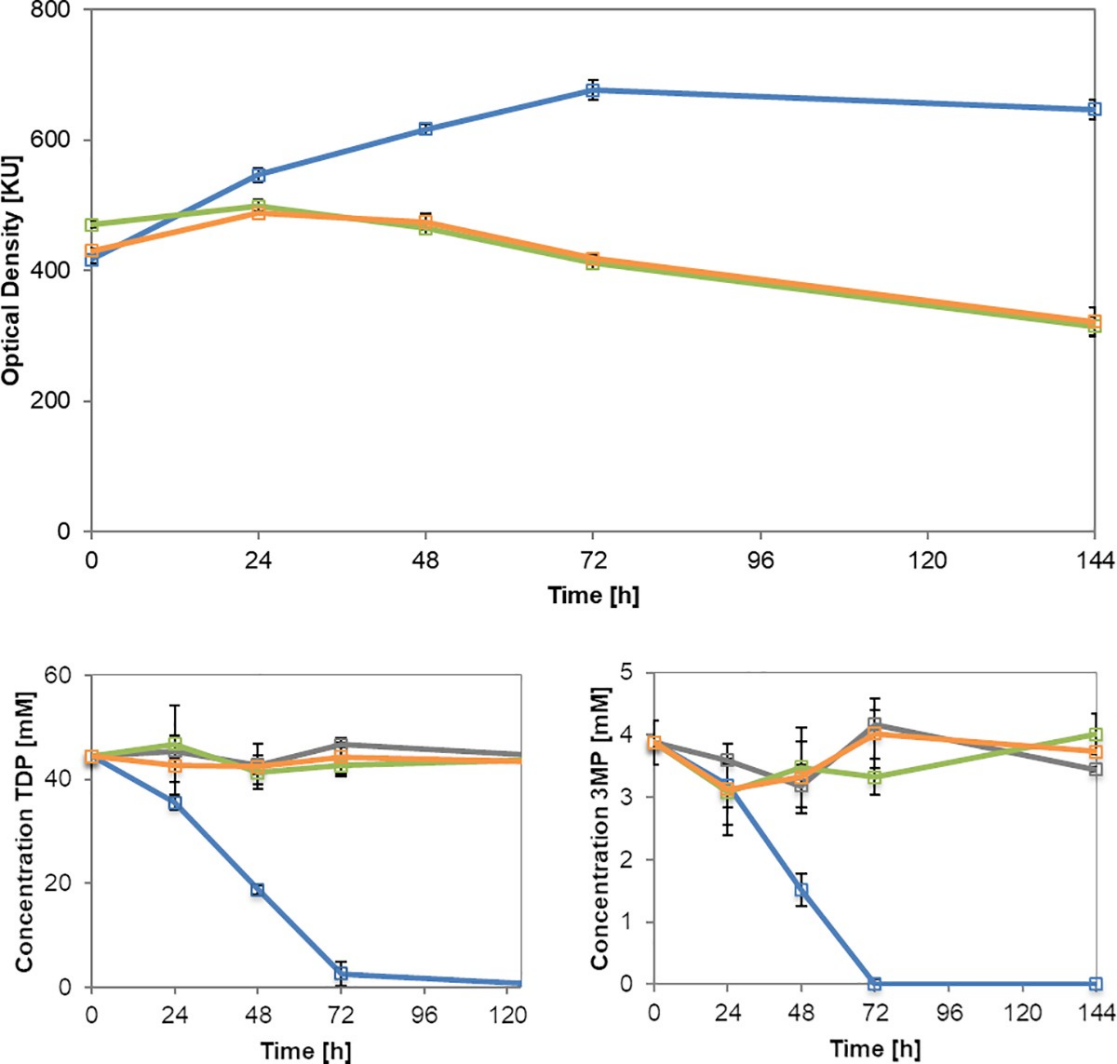
Supernatant analysis of the wildtype *V*. *paradoxus* TBEA6 in comparison to the mutants Δ*ech-20* and Δ*ech-30*. Cell mass was generated with MSM containing 60 mM succinate for 26 h (not displayed in the graph). Medium was exchanged after reaching 400 Klett Unit [KU]; subsequently, cells were grown with 10 mM succinate and 50 mM TDP. Samples were withdrawn at t = 0, 24, 48, 72, 144 h and analyzed via GC. *V*. *paradoxus* TBEA6 (blue), *V*. *paradoxus* TBEA6 Δ*ech-30* (green), *V*. *paradoxus* TBEA6 Δ*ech-20* (orange). Top: growth curve after medium exchange; bottom left: TDP-concentration in the supernatant detected via GC; bottom right: 3MP-concentration in the supernatant detected via GC.

In an experiment analyzing the metabolism of TDP by GC analysis of the substrate (Fig 5), the *ech* deletion mutants were cultivated in comparison to the wildtype. Both mutant strains and the wildtype were cultivated with succinate to a certain optical density. Then, cells were transferred to TDP-containing medium. Samples were withdrawn at different time points to analyze the consumption of TDP by the strains.

The growth curve after medium exchange showed how the wildtype cultures outstretched the exponential growth phase while both deletion mutants entered the stationary phase at 24 h to 48 h after medium exchange. At this time point, succinate (10 mM) was metabolized completely. The GC measurements exhibited a small amount of 3MP (4 mM) from the beginning of the experiment. Probably, TDP disintegrates in small amounts to 3MP due to cultivation conditions (temperature, oxygen). The wildtype immediately utilizes TDP and 3MP. The supernatant of both mutant cultures showed a decrease neither of TDP (45 mM) nor of 3MP (4 mM) until the end of the experiment as recorded for the cell-free controls. It can be concluded that TDP was not consumed by the *ech* deletion mutants. Consequently, Ech-20 and Ech-30 are involved in TDP degradation. Their genetic location (Fig 3) supports this hypothesis. The cluster comprises genes crucial for the metabolism of 3SP in TDP degradation: *mdo*, *act*, and *acdA* [1, 2, 17, 19].

**Fig 6:**
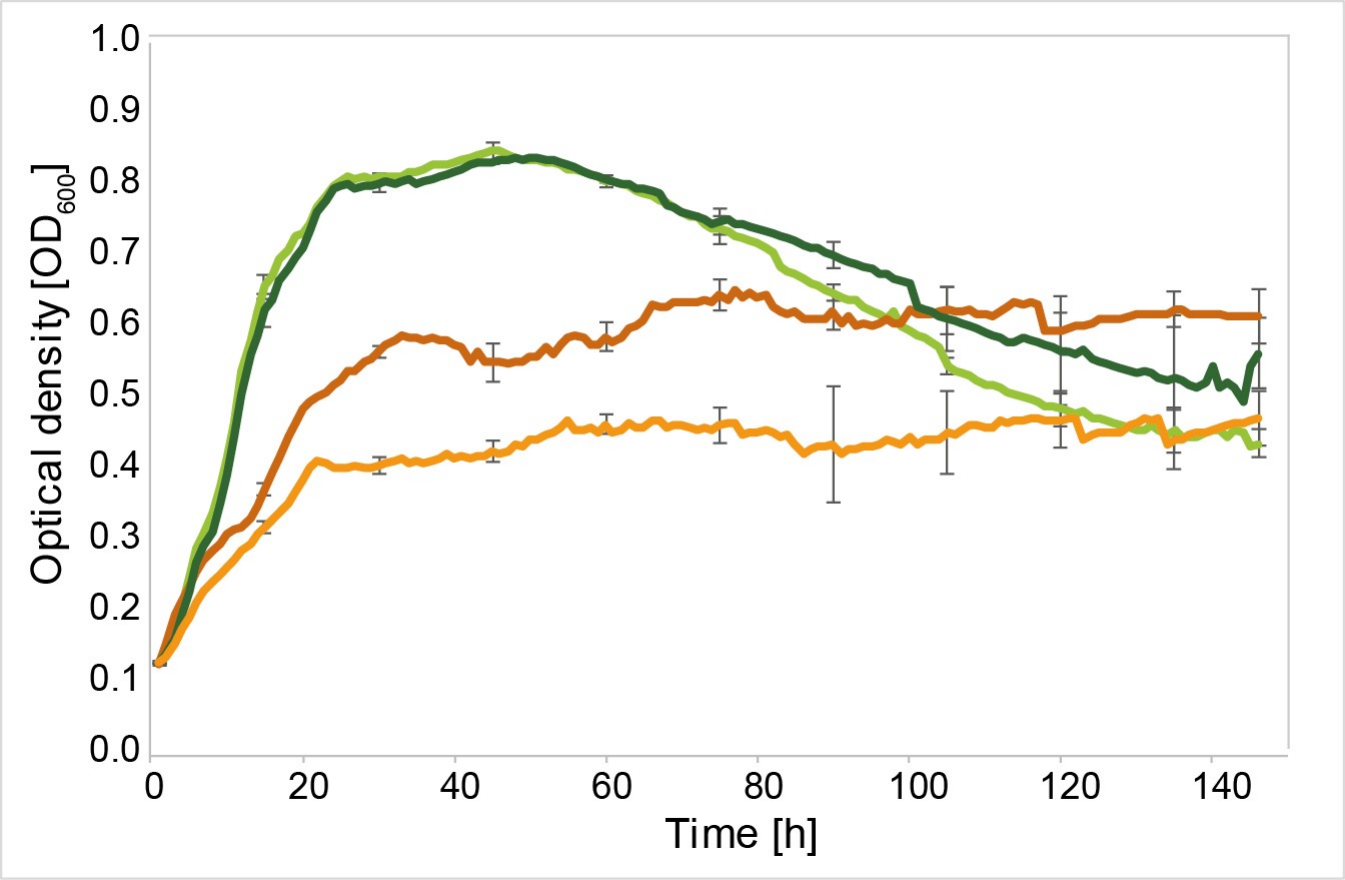
Growth of *V*. *paradoxus* TBEA6 and *V*. *paradoxus* Δ05510. Growth was monitored at 600 nm. Cells were cultivated in MSM containing 60 mM succinate (TBEA6 (dark green); Δ05510 (light green)) or 3,3´-thiodipropionate (TDP, TBEA6 (dark orange); Δ05510 (light orange)) as the only carbon source. The experiment was done in triplicate; standard deviations are indicated as error bars.

The deletion of another gene of this cluster, coding for a putative crotonase family protein (VPARA_05510), caused a reduced ability to consume TDP as observed in further cultivation experiments (Fig 6). Weak growth of the mutant with TDP as the exclusive carbon source indicated that this enzyme is also involved in the degradation of TDP.

**Fig 7:**
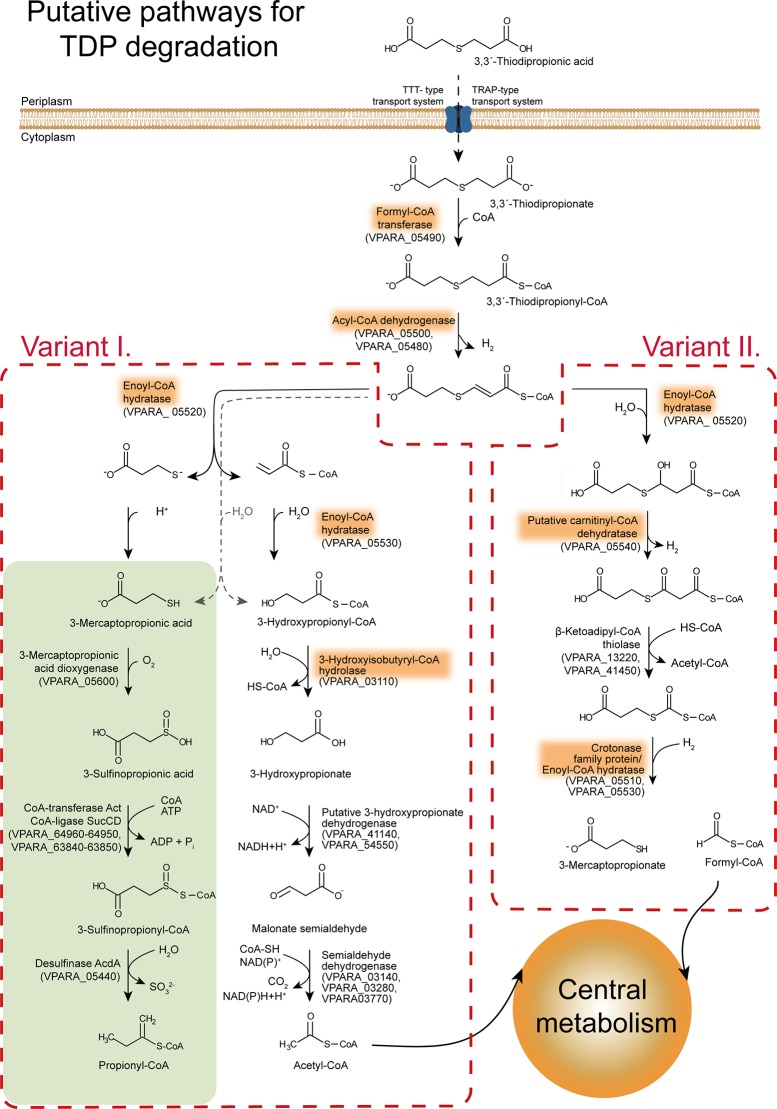
Hypothesis for the degradation of TDP. Possibly, TDP is converted to TDP-CoA by a putative formyl-CoA transferase. Presumably, a double bond is introduced to the molecule after the elimination of two protons by a putative acyl-CoA dehydrogenase. Afterward, two variants of the catabolism were postulated based on the protein profile from the proteome analysis. Variant I.: Degradation of TDP via formation of TDP-CoA and subsequent β-elimination resulting in the formation of acetyl-CoA by the enoyl-CoA hydratase homologs, the crotonase family protein homolog, a putative 3-hydroxypropionic acid dehydrogenase, and a semialdehyde dehydrogenase homolog. Variant II.: Degradation of TDP via β-oxidation resulting in the formation of formyl-CoA and 3-mercaptopropionate by the enoyl-CoA hydratases, a putative carnitinyl-CoA dehydratase, β-keto-thiolase homologs, and a putative crotonase family protein. The already described catabolism of 3-mercaptopropionate is shown in the grey box.

Based on these results, a preliminary pathway including Ech-20 and Ech-30 and the crotonase family protein was hypothesized. Participation of other enzymes and various possible reaction intermediates complicate the design of putative pathways (Fig 7) based on other publications. [37–42]. It is most likely that at least one acyl-CoA dehydrogenase plays a role during the process, since enoyl-CoA hydratases usually act on *trans*-enoyl-esters [43]. Some corresponding homologous genes are located in the gene cluster comprising important genes responsible for TDP degradation (Fig 3) (VPARA_05480 and VPARA_05500). Furthermore, acyl-CoA synthetases, hydroxyacyl-CoA dehydrogenases or ketoadipyl-CoA thiolases are possible participants.

**Fig 8:**
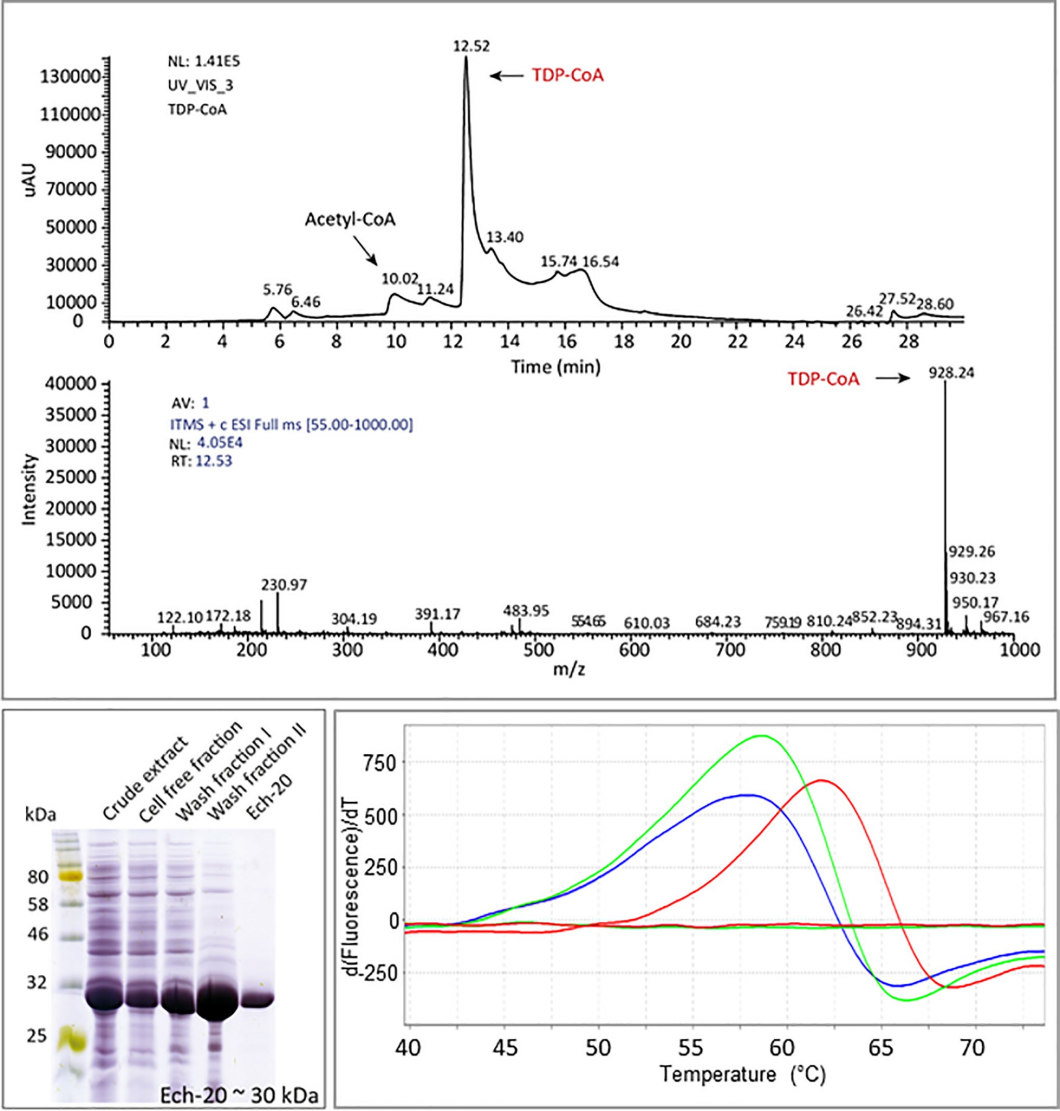
Synthesis of TDP-CoA and application in a thermal shift assay with Ech-20. A) Analysis of TDP-CoA via HPLC/MS using an AcclaimTM 120 C18 column. Detection was done at 259 nm with a photodiode array detector. TDP-CoA was detected at 12.52 min (upper diagram) and analyzed via mass spectrometry with 928.24 m/z (lower diagram). B) Heterologous production of Ech-20 in *E*. *coli* Bl21 (DE3) pLysS pET-32a(+)::*ech-20*. Purification of Ech-20 was performed via IMAC. 40 μg of protein were loaded onto the gel, except for elution samples of which only 5 μg were applied to the gel. C) Shift in the melting temperature of Ech-20 (2 μM) in presence of 2 mM TDP-CoA (red) and 2 mM TDP (green) shown in a thermal shift assay. The assay was performed with 2 μM protein in presence of 2 mM TDP-CoA in 100 mM Tris/HCl (pH 8). Control without protein (lines without peaks; green (TDP) and red (TDP-CoA)), control without ligand (blue).

Each pathway starts with the activation of TDP with CoA. Therefore, TDP-CoA was synthesized enzymatically employing the propionyl-CoA transferase of *Ralstonia eutropha* (Pct_Re_) (Fig 8). The purified TDP-CoA was subsequently used in thermal shift assays with Ech-20 and Ech-30 to elucidate a putative interaction of the enzymes and the CoA-ester. Thereby, a shift in the melting temperature of Ech-20 in the presence of TDP-CoA was detected (Fig 8) whereas no shift was observed in presence of TDP (Fig 8). This supports the assumption that activation of TDP with CoA is necessary for utilization by Ech-20. *In vitro* experiments are necessary to verify the enzyme’s impact in TDP-CoA hydrolysis. For Ech-30, no shift was detected with TDP-CoA or TDP (see supporting information, S4 Fig, S5 Fig). It is possible that this enzyme catalyzes a different step in TDP consumption. We also investigate the possibility that both enoyl-CoA hydratases might form a functional unit exhibiting a heterodimeric or heterotetrameric structure, as described by Yang et al. for a distinct MaoC-like enoyl-CoA hydratase from *Mycobacterium tuberculosis* [44]. This could be a reason why we can observe the interaction of TDP-CoA with Ech-20 but could not show any activity of the enzyme in a first attempt of an *in vitro* enzyme assay.

The presented findings confirm TDP-CoA as an intermediate of the TDP catabolism and delivered new insights into possible mechanisms of the TDP catabolism. Starting from TDP-CoA or its deprotonated form, one of the metabolic pathways of TDP could be the β-elimination-like pathway [40, 41] (Fig 7, variant I.). Ech-20 could mediate the first step and use TDP-CoA as a substrate. Regarding the missing interaction of Ech-30 with TDP-CoA, a reaction with another intermediate of the pathway is likely. Acrylyl-CoA would be the compound of choice, relating to variant I. of our postulated pathway. It is oxidized to 3HP-CoA which could be hydrolyzed to 3HP by the crotonase family protein. Variant II. would be similar to a β-oxidation-like pathway [38, 42, 45] (Fig 7, variant II.). TDP resembles the dicarboxylic acid pimelic acid [46], a substrate degraded via β-oxidation. Therefore, it is possible that TDP is catabolized via a similar pathway. Also on this route, one of the Ech (probably Ech-20) and the crotonase family protein could participate. The degradation products of this pathway would be 3MP and formyl-CoA. Different from the other variant, 3HP would not be an intermediate of TDP degradation. Further studies are necessary to reinforce our hypotheses, unravel the function of the remaining enzymes (e.g. hydroxybutyryl-CoA hydrolase), and determine the actual pathway.

## Conclusion

This study showed that TDP consumption affects several different cellular processes. Therefore, many of the corresponding proteins showed a high abundance during proteome analysis. Genes encoding high abundant proteins identified by proteome analysis were deleted. Thereby, three mutants lacking a crotonase family protein (VPARA_05510) or one of two Echs (VPARA_05520 and VPARA_05530) exhibited impaired growth using TDP as the only source of carbon. Further investigations such as growth experiments and supernatant analysis of the mutants as well as the stabilization of Ech-20 with TDP-CoA confirmed the results from gene deletion. We are now able to postulate the significance of Ech-20 of *V*. *paradoxus* TBEA6 for conversion of TDP to 3MP. Also for Ech-30 and the crotonase family protein, we were able to assign a potential function. Comparing the new pathway with the formerly postulated one, we gained new insights into the catabolism of TDP, its modification, and further degradation.

## Supporting information

S1 Table Primers used for generation of suicide plasmids for marker-free gene deletion. The designation contains the locus tag, downstream (Do) or upstream (Up) location of the respective flanking sequence, and forward (FOR) or reverse (REV) orientation of the primer. Furthermore, melting temperatures (T_M_) are included in the table. Restriction site are inserted into the primer in front of the sequence for cloning (underlined).
(PDF)

S2 Table Primers used for verification of marker-free gene deletion. The designation contains the locus tag, binding of the primer inside (In) or outside (Ex) of the deleted gene, and forward (FOR) or reverse (REV) orientation of the primer. Furthermore, melting temperatures (T_M_) are included in the table.
(PDF)

S3 Table Quantitation table of the first biological experiment. Displayed are the normalized and mean normalized spot volumes of spots on gels from cultivations with gluconate in contrast to TDP and 3SP, ratios of mean normalized volumes, spot labels, and detected proteins (MALDI-TOF-MS/MS) within respective spots (Accession number). Some accession numbers are missing for spots that were not identified via MALDI-TOF-MS/MS.
(PDF)

S4 Table Quantitation table of the second biological experiment. Displayed are the normalized and mean normalized spot volumes of spots on gels from cultivations with gluconate in contrast to TDP and 3SP, ratios of mean normalized volumes, and detected proteins (MALDI-TOF-MS/MS) within respective spots (Accession number). Some accession numbers are missing for spots that were not identified via MALDI-TOF-MS/MS. Identified spots from the already described TDP metabolism are highlighted in blue; protein spots that belong to genes deleted during this study are highlighted in orange; proteins from both, which were found in the same spot, are marked in grey.
(PDF)

S1 Fig 2D-gels from the second biological experiment with spot labels. Cells cultivated with Gluconate and TDP in comparison were disrupted and proteins extracted for 2D-gel analysis. Proteins (1.5 μg per gel) were firstly separated by isoelectric point (pH 5 to pH 8) and secondly by molecular weight. Proteins were stained with Coomassie Brilliant Blue, scanned, labeled, and analyzed via the Delta2D Software. Spot labels are displayed on the fused images.
(PDF)

S2 Fig 2D-gels from the second biological experiment with spot labels. Cells cultivated with Gluconate and 3SP in comparison were disrupted and proteins extracted for 2D-gel analysis. Proteins (1.5 μg per gel) were firstly separated by isoelectric point (pH 5 to pH 8) and secondly by molecular weight. Proteins were stained with Coomassie Brilliant Blue, scanned, labeled, and analyzed via the Delta2D Software. Spot labels are displayed on the fused images.
(PDF)

S3 Fig 2D-gels from the second biological experiment with spot labels. Cells cultivated with TDP and 3SP in comparison were disrupted and proteins extracted for 2D-gel analysis. Proteins (1.5 μg per gel) were firstly separated by isoelectric point (pH 5 to pH 8) and secondly by molecular weight. Proteins were stained with Coomassie Brilliant Blue, scanned, labeled, and analyzed via the Delta2D Software. Spot labels are displayed on the fused images.
(PDF)

S4 Fig Purification of Ech-30. Heterologous expression was performed using cells of *E*. *coli* BL21 (DE3) pLysS which were transformed with pET23a::*ech*-30. ZYP autoinduction medium was inoculated with freshly transformed cells and the cultures were incubated overnight at 30°C on a rotary shaker at 130 rpm. Purification was achieved using His Spin Trap columns (GE Healthcare) using 100 mM Tris/HCl buffer (pH 8.0 with 500 mM NaCl and different concentrations of imidazole. Cells were mixed with binding buffer (20 mM imidazole) before cell disruption and afterwards cell debris was removed by centrifugation (cell-free lysate). The cell-free lysate was loaded onto the columns (flow through) and washed with the above mentioned Tris/HCl Buffer with the following concentrations of imidazole: wash fraction I, 50 mM imidazole; wash fraction II, 100 mM imidazole; wash fraction III and IV, 200 mM imidazole. The protein was eluted using the Tris/HCl buffer with 500 mM imidazole.
(PDF)

S5 Fig Thermal shift assay of purified Ech-30 using TDP (green) and TDP-CoA (red) as ligands. The blue curve represents the no ligand control. The lines without the peaks represent the no protein controls, also with addition of TDP (green) and TDP-CoA (red). The assay was performed in triplicate with 2 μM protein in presence of 2 mM TDP-CoA in 100 mM Tris/HCl (pH 8).
(PDF)
