# Supplementary material for: The catabolism of 3,3’-thiodipropionic acid in Variovorax paradoxus strain TBEA6: A proteomic analysis
Source: PLoS One. 2019 Feb 11;14(2):e0211876. doi: 10.1371/journal.pone.0211876 (PMC6370202; doi:10.1371/journal.pone.0211876)
Supplement: S1 Table — The designation contains the locus tag, downstream (Do) or upstream (Up) location of the respective flanking sequence, and forward (FOR) or reverse (REV) orientation of the primer. Furthermore, melting temperatures (TM) are included in the table. Restriction site are inserted into the primer in front of the sequence for cloning (underlined). (PDF) [file pone.0211876.s001.pdf]

S1 Table: Primers used for generation of suicide plasmids for marker-free gene deletion. The designation contains the locus tag, downstream (Do) or upstream (Up) location of the respective flanking sequence, and forward (FOR) or reverse (REV) orientation of the primer. Furthermore, melting temperatures (T_M) are included in the table. Restriction site are inserted into the primer in front of the sequence for cloning (underlined).

Designation upstream primer	Sequence (5'-xxx-3')	T_M [°C]	Designation downstream primer	Sequence (5'-xxx-3')	T_M [°C]
05510 Up Fl FOR	aaaatctagaGATTCCCGAAGAAGCTGACCTACG	62,4	05510 Do Fl FOR	aaaagaattcCGTCAGAGACATTCAACGCGTCCGAGG	61.9
05510 Up Fl REV	aaaagaattcTTTGTACTTTGCCAAAGAGAGAGTGAGTG	64	05510 Do Fl REV	aaaatctagaCAGCGCCGAAGCCACGGCGTTCC	62.2
05520 Up Fl FOR	aaaatctagaCTCGCCGTTCCCTGCTCGAAG	62.8	05520 Do Fl FOR	aaaagaattcCTCCCGCACTCACTCTCTCTTTGG	60.2
05520 Up Fl REV	aaaagaattcTTGCCGCGGAACACAGGCTTG	64.7	05520 Do Fl REV	aaaatctagaGTGAATATCATTGCCGCGCAAC	59.0
05530 Up Fl FOR	aaaatctagaCTGACGCACGTTTCTCAGGCG	60.4	05530 Do Fl FOR	aaaagaattcGGGGCGCAACCGTGAAGATC	61.1
05530 Up Fl REV	aaaagaattcAGTTTGTCTTCCTGTGTTCGAGTGAGAG	59.4	05530 Do Fl REV	aaaatctagaCTCGTTCTGCTTCTCGCCGC	59.7
05540 Up Fl FOR	aaaatctagaCCGTGCTTGCTCCACAGTCATTAAAC	61.7	05540 Do Fl FOR	aaaaggatccGACAGAATTCGCCGCGCG	59.9
05540 Up Fl REV	aaaaggatccTCGTATCTCCATTTTTCAATGACAAGGG	61.2	05540 Do Fl REV	aaaatctagaGTTCACCGATGCCGCAGGTG	60.8
05550 Up Fl FOR	aaaatctagaGCGACCTGCTCATTGCTGCG	61.3	05550 Do Fl FOR	aaaagagctcTCCGGCGCTTTGGCCG	61.2
05550 Up Fl REV	aaaagagctcATTTGAGTACTCTGAGATGGGAGCCCAC	61.8	05550 Do Fl REV	aaaatctagaGACCGAACGCACATAGCCGC	60.1
15130 Up Fl FOR	aaaatctagaCGGTCGGTCATGGCCTTGAG	59.9	15130 Do Fl FOR	aaaagaattcTGCGCGCTGATCAATTGCTG	60.4
15130 Up Fl REV	aaaagaattcGAGAACTCCAACAAAACTAGTTTGAAAGGTG	60.1	15130 Do Fl REV	aaaatctagaCTGCGTCTCCTAAGAACCGTTCG	59.0
19450 Up Fl FOR	aaaatctagaCTGCAGGACATCGGCATCACC	59.9	19450 Do Fl FOR	aaaagaattcGTAAGCTGCGTGCCTGCTTCC	58.7
19450 Up Fl REV	aaaagaattcGGCGAGAGACTCCTAAAAATCAAAAAG	58.3	19450 Do Fl REV	aaaatctagaGTGCGCTTTCCGGTATTTTCG	58.3
21730 Up Fl FOR	aaaatctagaCATTGCAGGCCCGCAAGATG	60.9	21730 Do Fl FOR	aaaagaattcTCCCTTCGGAAGCGCCC	58.2
21730 Up Fl REV	aaaagaattcCGGGCGTCTCCTGGGGTG	60.8	21730 Do Fl REV	aaaatctagaGGCTGGTACACCATCCTCACCG	60.1
24490 Up Fl FOR	aaaatctagaGCAGCGAGATCTCGGCGTTC	59.5	24490 Do Fl FOR	aaaagaattcTCGAGCCCGCCACCATGGA	64.0
24490 Up Fl REV	aaaagaattcTGCCGTTTTTCTCCGTTCCG	59.5	24490 Do Fl REV	aaaatctagaCACGGCTTCAGACCTTCCAGAC	57.5
24900 Up Fl FOR	aaaatctagaGATGATGTCGATGAGTACCAAGGAAAAC	59.2	24900 Do Fl FOR	aaaaggatccGGCTCGCCCCCCCCAGG	64.2
24900 Up Fl REV	aaaaggatccGTCGAACGCTCCTTGCGTCAATG	62.5	24900 Do Fl REV	aaaatctagaCTATTACTTCGGGGCGCTGACCG	62.5
27740 Up Fl FOR	aaaatctagaGCTGCCGCCCGTGAAATAGCG	65.2	27740 Do Fl FOR	aaaagaattcCTCCACCAGGACCTCTATGCCC	58.5
27740 Up Fl REV	aaaagaattcGCAGAAGGCGGGTGCCCCTG	65.4	27740 Do Fl REV	aaaatctagaCCACGCCGGTATCGACCAC	58.3
27760 Up Fl FOR	aaaagagctcGCTTCGCCCAGGCGAAGTG	60.3	27760 Do Fl FOR	aaaagaattcACCAGGGCCGTCCGGCCGTG	69.5
27760 Up Fl REV	aaaagaattcTTGGCAAATTCCTCTCAAGTCGAGTC	60.7	27760 Do Fl REV	aaaagagctcCTTCGAAGAAGACATCGGACCCGAGCC	68.1
34530 Up Fl FOR	aaaatctagaCATCTGTCGGCAGGCCAGAAC	59.2	34530 Do Fl FOR	aaaagaattcAACCGACAATGGAATCCTTCCC	57.8
34530 Up Fl REV	aaaagaattcGGCGAGTTGGCGTTCCGG	60.9	34530 Do Fl REV	aaaatctagaCCGGATCAATGGGCTTGCC	59.7
