# Supplementary material for: The catabolism of 3,3’-thiodipropionic acid in Variovorax paradoxus strain TBEA6: A proteomic analysis
Source: PLoS One. 2019 Feb 11;14(2):e0211876. doi: 10.1371/journal.pone.0211876 (PMC6370202; doi:10.1371/journal.pone.0211876)
Supplement: S2 Table — The designation contains the locus tag, binding of the primer inside (In) or outside (Ex) of the deleted gene, and forward (FOR) or reverse (REV) orientation of the primer. Furthermore, melting temperatures (TM) are included in the table. (PDF) [file pone.0211876.s002.pdf]

S2 Table: Primers used for verification of marker-free gene deletion. The designation contains the locus tag, binding of the primer inside (In) or outside (Ex) of the deleted gene, and forward (FOR) or reverse (REV) orientation of the primer. Furthermore, melting temperatures (T_M) are included in the table.

Designation internal primer	Sequence (5‘-xxx-3‘)	T_M [°C]	Designation external primer	Sequence (5‘-xxx-3‘)	T_M [°C]
05510 In FOR	CTATGCTGCGGAAGTGGGCC	63.5	05510 Ex FOR	CGCCTCGTCTCGACCCTTTATC	64
05510 In REV	CCGGTCCTTGTCGATGAGGA‘	63.7	05510 Ex REV	CGGCTCGATCCAGTGCTGC	63.1
05520 In FOR	GGGCGCAACCGTGAAGATC	57.6	05520 Ex FOR	CTTCAGCGTGGGCTCCGATG	60.2
05520 In REV	CATTCAGCCAGGCAGGCAG	56.5	05520 Ex REV	GTCGTCGGTGTTGAAATCGGC	58.9
05530 In FOR	GATCGTGCCAAGCAGAAGAATGC	59.7	05530 Ex FOR	GGCGCGTCCTGCCGAAC	59.7
05530 In REV	GTAGAACGATTCCATAGGGCTTGTTG	58.3	05530 Ex REV	GTGCAGCAGGTGATCGAGTTCGTAC	60.8
05540 In FOR	GATGTCGTTACCTATGAGTCCGCC	57.7	05540 Ex FOR	GAGCCCGCTCACATGAGGTCG	61.4
05540 In REV	GTTGACCAACCGGCACGATCC	60.9	05540 Ex REV	GTCTTCTTCCCGTTGCGCAGG	60.5
05550 In FOR	CGACGCGCCGTTGACTTCG	62.1	05550 Ex FOR	GGGAGGCGCCATGGTTCTTG	61.1
05550 In REV	CTGCAAGGCGACTACTTTGCGATC	61.0	05550 Ex REV	GCAGCCCCCGCGCTGTAG	61.2
15130 In FOR	GAAGGCGCAGCCGCTCAAG	60.5	15130 Ex FOR	CGCCTGGAGGTCCTTTTGCC	60.4
15130 In REV	CATCTCGTTCAGGCGGTGCAC	60.1	15130 Ex REV	CGTAGGTCACCGAGCAGAACTCG	59.8
19450 In FOR	CGAAATCCGCGATGTCGATCTCTAC	61.8	19450 Ex FOR	GGACAAGGCGCCCAACAAGAC	60.3
19450 In REV	GCTGAGGTCAATGCCGTAATAGTCG	59.9	19450 Ex REV	GCGGATACCACTGCCCACCG	61.9
21730 In FOR	GGTGCAAGGCGGCATCAGC	61.1	21730 Ex FOR	CTTCGACGCGTCGATCTTGC	58.1
21730 In REV	CACGCAGGTGTCGGTCGAGATC	61.3	21730 Ex REV	GCGAAAGCAGCCAGCTCGAG	59.8
24490 In FOR	GAATACATCCCGGTGCATATTGCC	60.1	24490 Ex FOR	CTGTAGATCAGGTCGCCGGGC	60.4
24490 In REV	GCTGAAGTCGCAGTCGAAGCG	59.7	24490 Ex REV	GCACTGGTGGTTTACCTGATGGG	59.1
24900 In FOR	CACTCCCTCCGGCCTGCAATAC	61.4	24900 Ex FOR	GCCCTGCACAGGATCGATGG	59.8
24900 In REV	GTCGAGCAGCTCGACGTCGAAC	60.9	24900 Ex REV	GATAACCCGGCGCGATTCAAG	60.2
27740 In FOR	CGAGTGGCGCCGCACCTAC	61.1	27740 Ex FOR	GCTCCTCGCGCGTGATGG	59.9
27740 In REV	GGCGACGGCGATGTCGTG	60.6	27740 Ex REV	CGCTACTGCGTGGAATTCCTTG	58.5
27760 In FOR	GTGGCCGCGCACATCGAG	60.7	27760 Ex FOR	GTAGCGGTGGCGTGGAACAGG	61.2
27760 In REV	CGCGGTGCCATTGGCATC	60.4	27760 Ex REV	GCACACAATCTGCACCCATGAGATC	61.4
34530 In FOR	CTGCTGTGCCCCTATGTGCAGC	61.4	34530 Ex FOR	GACGTGCTGCTCTCCTCGACCC	61.9
34530 In REV	GCGCCAGGCATTCACCTTG	62.1	34530 Ex REV	CGGAATGACGCCGACAATCAG	59.9
