# Supplementary material for: The catabolism of 3,3’-thiodipropionic acid in Variovorax paradoxus strain TBEA6: A proteomic analysis
Source: PLoS One. 2019 Feb 11;14(2):e0211876. doi: 10.1371/journal.pone.0211876 (PMC6370202; doi:10.1371/journal.pone.0211876)
Supplement: S3 Table — Displayed are the normalized and mean normalized spot volumes of spots on gels from cultivations with gluconate in contrast to TDP and 3SP, ratios of mean normalized volumes, spot labels, and detected proteins (MALDI-TOF-MS/MS) within respective spots (Accession number). Some accession numbers are missing for spots that were not identified via MALDI-TOF-MS/MS. (PDF) [file pone.0211876.s003.pdf]

S3 Table: Quantitation table of the first biological experiment. Displayed are the normalized and mean normalized spot volumes of spots on gels from cultivations with gluconate in contrast to TDP and 3SP, ratios of mean normalized volumes, spot labels, and detected proteins (MALDI-TOF-MS/MS) within respective spots (Accession number). Some accession numbers are missing for spots that were not identified via MALDI-TOF-MS/MS.

Spot number	Normalized Volume Gluc (Vol%)			Mean Norm. Volume Gluc (Vol%)	Normalized Volume TDP (Vol%)			Mean Norm. Volume TDP (Vol%)	Normalized Volume 3SP (Vol%)			Mean Norm. Volume 3SP (Vol%)	Ratio of mean Norm. Volumes (Vol%/Vol%) TDP / Gluc	3SP / Gluc	TDP / 3SP	Accession numbers (VPARA_XXXXX)	
4	0.017	0.009	0.023	0.016	0.025	0.040	0.033	0.032	0.088	0.074	0.114	0.092	2.003	5.675	0.353		
5	0.004	0.003	0.023	0.010	0.005	0.039	0.025	0.023	0.063	0.049	0.055	0.055	2.342	5.587	0.419		
6	0.023	0.005	0.020	0.016	0.005	0.022	0.024	0.017	0.067	0.056	0.064	0.062	1.054	3.832	0.275		
12	0.374	0.434	0.293	0.367	0.222	0.206	0.290	0.239	0.203	0.049	0.040	0.097	0.652	0.265	2.456	45650	
19	0.134	0.006	0.012	0.051	0.333	0.376	0.215	0.308	0.000	0.039	0.066	0.035	6.077	0.690	8.805		
24	0.103	0.084	0.083	0.090	0.205	0.182	0.154	0.180	0.173	0.182	0.213	0.189	2.006	2.105	0.953		
31	0.152	0.253	0.279	0.228	0.151	0.116	0.131	0.133	0.031	0.007	0.031	0.023	0.582	0.100	5.817		
32	0.172	0.046	0.118	0.112	0.498	0.615	0.578	0.563	0.481	0.456	0.080	0.339	5.020	3.020	1.662		
33	0.173	0.097	0.148	0.139	0.145	0.054	0.111	0.103	0.062	0.048	0.031	0.047	0.741	0.335	2.210	05540	
38	0.374	0.289	0.343	0.335	3.070	2.710	2.400	2.727	4.447	5.708	4.677	4.944	8.131	14.742	0.552	38990	
45	0.555	0.903	0.757	0.738	0.424	0.539	0.409	0.457	0.013	0.144	0.208	0.122	0.620	0.165	3.755	05510	
46	0.001	0.011	0.002	0.005	0.000	0.016	0.000	0.006	0.070	0.029	0.021	0.040	1.152	8.324	0.138	05450	
47	0.139	0.080	0.220	0.146	0.499	0.303	0.384	0.395	1.371	0.292	0.590	0.751	2.704	5.142	0.526	39020	
49	0.071	0.046	0.011	0.043	0.232	0.237	0.257	0.242	0.623	0.148	0.489	0.420	5.670	9.849	0.576	11790	27740
50	0.013	0.017	0.006	0.012	0.029	0.019	0.005	0.017	0.067	0.060	0.021	0.050	1.456	4.133	0.352		
51	0.001	0.007	0.002	0.004	0.041	0.041	0.034	0.038	0.218	0.136	0.189	0.181	10.776	50.728	0.212	61040	03000
52	0.001	0.017	0.023	0.014	0.000	0.100	0.024	0.041	0.193	0.154	0.153	0.167	3.015	12.136	0.248	04450	
53	0.227	0.058	0.077	0.121	1.746	0.523	1.029	1.099	2.714	1.032	1.604	1.784	9.090	14.748	0.616	04890	

54	0.000	0.007	0.006	0.004	0.000	0.020	0.003	0.008	0.058	0.027	0.012	0.033	1.756	7.578	0.232		
55	0.015	0.000	0.002	0.006	0.117	0.040	0.040	0.066	0.021	0.002	0.000	0.008	11.703	1.355	8.634	04890	
56	0.044	0.010	0.081	0.045	0.077	0.053	0.039	0.056	0.123	0.146	0.119	0.129	1.254	2.885	0.435		
58	0.123	0.048	0.128	0.100	0.103	0.104	0.105	0.104	0.158	0.243	0.213	0.205	1.043	2.051	0.508	05600	
59	0.024	0.011	0.013	0.016	0.026	0.033	0.028	0.029	0.045	0.051	0.057	0.051	1.803	3.156	0.571	25880	
62	0.265	0.345	0.152	0.254	0.698	0.525	0.530	0.584	0.331	0.204	0.304	0.280	2.299	1.100	2.090	11100	03010
66	0.170	0.195	0.065	0.143	0.420	0.408	0.392	0.407	0.282	0.147	0.301	0.243	2.835	1.694	1.673	05550	
67	0.126	0.126	0.049	0.100	0.264	0.279	0.258	0.267	0.104	0.140	0.116	0.120	2.660	1.198	2.220	38040	
72	0.097	0.089	0.051	0.079	0.019	0.028	0.030	0.025	0.003	0.012	0.006	0.007	0.320	0.093	3.431		
73	0.489	0.286	0.711	0.495	1.769	1.284	1.765	1.606	2.388	1.854	1.928	2.057	3.244	4.155	0.781	05550	05520
75	0.152	0.085	0.118	0.118	0.569	0.436	0.581	0.529	0.503	0.444	0.377	0.441	4.465	3.726	1.198	27730	05550
76	0.868	0.321	0.607	0.599	0.446	0.469	0.498	0.471	0.279	0.223	0.274	0.259	0.786	0.432	1.819		
81	0.174	0.039	1.038	0.417	0.228	0.227	0.171	0.209	0.008	0.099	0.104	0.070	0.500	0.168	2.977		
82	0.000	0.000	0.001	0.000	0.134	0.111	0.150	0.131	0.024	0.018	0.040	0.027	434.060	90.250	4.810		
84	0.118	0.135	0.210	0.155	0.232	0.164	0.192	0.196	0.356	0.523	0.384	0.421	1.267	2.725	0.465	27730	
87	0.026	0.021	0.025	0.024	0.034	0.029	0.024	0.029	0.019	0.003	0.013	0.012	1.208	0.483	2.500		
88	0.432	0.800	0.522	0.585	0.284	0.302	0.267	0.284	0.138	0.087	0.162	0.129	0.486	0.221	2.203	05440	
89	0.201	0.301	0.564	0.355	0.676	1.316	0.792	0.928	0.507	0.233	0.157	0.299	2.611	0.841	3.104	05440	
91	0.884	0.972	0.954	0.937	1.931	2.915	2.449	2.432	1.091	0.927	1.577	1.198	2.595	1.279	2.029	05440	
96	0.336	0.376	0.330	0.347	0.194	0.214	0.171	0.193	0.152	0.048	0.062	0.087	0.556	0.252	2.207	19070	
101	0.254	0.155	0.370	0.260	0.687	1.241	1.149	1.026	0.480	0.279	0.301	0.353	3.946	1.359	2.904	05440	
104	0.149	0.181	0.178	0.169	0.059	0.071	0.035	0.055	0.040	0.003	0.001	0.015	0.326	0.087	3.742	05540	05540
105	0.474	0.493	0.487	0.485	0.610	0.542	0.638	0.597	1.055	1.047	1.401	1.168	1.231	2.408	0.511	05510	
106	0.065	0.057	0.107	0.076	0.071	0.097	0.136	0.101	0.256	0.184	0.349	0.263	1.331	3.455	0.385		
108	0.309	0.282	0.312	0.301	0.454	0.434	0.538	0.475	0.875	0.853	0.871	0.866	1.579	2.877	0.549	05770	
109	0.186	0.199	0.158	0.181	0.256	0.263	0.346	0.288	0.440	0.407	0.476	0.441	1.591	2.435	0.653		
110	0.573	0.413	0.439	0.475	0.832	0.810	0.932	0.858	1.407	1.274	1.658	1.446	1.805	3.044	0.593	05770	
111	0.246	0.266	0.272	0.261	0.552	1.159	0.671	0.794	0.753	0.355	0.425	0.511	3.039	1.956	1.554		
112	0.243	0.256	0.286	0.262	0.749	0.820	0.925	0.832	1.094	0.874	1.045	1.004	3.178	3.839	0.828	05450	
113	0.060	0.042	0.087	0.063	0.098	0.123	0.100	0.107	0.158	0.166	0.311	0.212	1.693	3.349	0.505		

114	0.271	0.271	0.261	0.268	1.246	1.904	1.729	1.626	1.186	0.923	1.223	1.110	6.073	4.148	1.464	05450		
115	0.107	0.160	0.137	0.135	0.603	0.507	0.499	0.536	0.623	0.388	0.462	0.491	3.981	3.644	1.093	05490		
116	0.033	0.041	0.039	0.038	0.177	0.189	0.214	0.193	0.207	0.138	0.199	0.181	5.155	4.834	1.066	05490		
117	0.058	0.095	0.078	0.077	0.470	0.430	0.464	0.455	0.451	0.350	0.417	0.406	5.908	5.277	1.120			
119	0.016	0.037	0.018	0.024	0.075	0.068	0.077	0.073	0.017	0.027	0.007	0.017	3.107	0.718	4.329	45870	03010	
121	0.141	0.116	0.120	0.126	0.243	0.287	0.346	0.292	0.221	0.187	0.236	0.214	2.325	1.707	1.362	05550		
123	0.095	0.087	0.105	0.096	0.178	0.188	0.225	0.197	0.123	0.084	0.117	0.108	2.058	1.132	1.817	12060		
124	0.077	0.076	0.060	0.071	0.219	0.286	0.240	0.248	0.257	0.180	0.278	0.238	3.499	3.355	1.043			
125	0.072	0.133	0.104	0.103	0.212	0.215	0.260	0.229	0.317	0.181	0.245	0.248	2.222	2.405	0.924	05550		
126	0.341	0.222	0.262	0.275	0.304	0.243	0.360	0.302	0.115	0.076	0.159	0.117	1.098	0.425	2.581			
127	0.177	0.271	0.248	0.232	0.004	0.088	0.054	0.049	0.018	0.015	0.019	0.017	0.209	0.075	2.800	05530		
128	0.129	0.117	0.102	0.116	0.056	0.048	0.030	0.045	0.046	0.003	0.018	0.022	0.384	0.194	1.983			
135	0.203	0.192	0.110	0.168	0.115	0.127	0.076	0.106	0.048	0.033	0.064	0.049	0.627	0.289	2.171	05540		
137	0.311	0.226	0.387	0.308	0.924	1.106	1.214	1.081	0.106	0.090	0.151	0.116	3.510	0.377	9.320	05540		
139	0.038	0.001	0.035	0.025	0.232	0.202	0.248	0.227	0.034	0.013	0.054	0.034	9.229	1.375	6.710	03330		
142	0.133	0.115	0.111	0.119	0.063	0.049	0.047	0.053	0.017	0.018	0.051	0.028	0.447	0.239	1.871			
144	0.058	0.023	0.073	0.051	0.269	0.291	0.306	0.288	0.137	0.118	0.133	0.129	5.643	2.529	2.231	27730		
146	0.487	0.653	0.509	0.550	1.205	1.294	1.742	1.414	2.150	1.916	2.081	2.049	2.571	3.727	0.690	14190		
147	0.157	0.172	0.191	0.173	0.403	0.469	0.595	0.489	0.649	0.600	0.614	0.621	2.821	3.579	0.788			
148	0.661	0.748	0.528	0.646	1.624	2.084	1.777	1.828	2.221	1.843	2.200	2.088	2.831	3.233	0.876	14190		
151	0.047	0.090	0.084	0.074	0.275	0.291	0.388	0.318	0.455	0.419	0.360	0.411	4.309	5.572	0.773			
152	0.046	0.061	0.049	0.052	0.170	0.154	0.282	0.202	0.332	0.304	0.312	0.316	3.902	6.104	0.639	24880		
153	0.121	0.149	0.106	0.125	0.383	0.397	0.366	0.382	0.552	0.414	0.404	0.457	3.053	3.647	0.837	24880		
155	0.260	0.425	0.107	0.264	0.728	0.903	0.697	0.776	0.785	0.556	0.790	0.710	2.938	2.690	1.092	59130	11100	19450
156	0.286	0.274	0.111	0.224	0.541	0.948	0.522	0.670	0.659	0.260	0.121	0.347	2.996	1.550	1.933	09890		
157	0.274	0.335	0.303	0.304	0.622	0.841	0.786	0.750	0.478	0.406	0.326	0.403	2.465	1.326	1.859	67900		
161	1.052	1.694	0.973	1.239	1.717	2.277	2.338	2.111	2.171	2.530	2.808	2.503	1.703	2.020	0.843	03000		
164	0.228	0.204	0.256	0.229	0.056	0.085	0.082	0.074	0.068	0.038	0.083	0.063	0.324	0.276	1.172	05510	05440	
167	0.033	0.012	0.059	0.035	0.114	0.139	0.099	0.117	0.248	0.145	0.131	0.175	3.391	5.048	0.672			
168	0.350	0.212	0.292	0.284	0.094	0.063	0.064	0.074	0.020	0.022	0.014	0.019	0.259	0.065	3.971			

169	0.114	0.128	0.174	0.139	0.061	0.074	0.078	0.071	0.037	0.045	0.021	0.034	0.512	0.248	2.063	42940	
172	0.184	0.233	0.184	0.200	0.085	0.084	0.128	0.099	0.114	0.074	0.065	0.084	0.494	0.422	1.172		
180	0.067	0.047	0.057	0.057	0.268	0.202	0.317	0.262	0.112	0.043	0.041	0.065	4.616	1.152	4.006	03280	
181	0.713	0.572	0.572	0.619	2.032	2.092	2.223	2.116	1.300	0.851	0.935	1.029	3.417	1.662	2.056	03010	
183	0.353	0.356	0.302	0.337	1.326	3.809	2.145	2.427	1.341	2.281	1.423	1.681	7.199	4.988	1.443	04900	
186	0.657	0.832	0.732	0.740	4.774	4.699	6.270	5.248	4.685	4.236	4.382	4.434	7.089	5.990	1.183	04900	05450
187	0.246	0.234	0.210	0.230	0.764	0.627	0.992	0.795	0.943	0.826	1.075	0.948	3.459	4.126	0.838		
190	0.211	0.273	0.266	0.250	0.482	0.745	0.720	0.649	0.697	0.763	0.806	0.755	2.595	3.019	0.859		
192	0.222	0.309	0.299	0.276	1.790	1.642	1.819	1.750	2.324	2.375	2.327	2.342	6.332	8.472	0.747	04650	
198	0.027	0.006	0.001	0.011	0.095	0.098	0.100	0.098	0.032	0.032	0.043	0.036	8.741	3.196	2.735		
203	0.155	0.243	0.112	0.170	0.071	0.091	0.028	0.064	0.019	0.006	0.003	0.009	0.374	0.055	6.754		
206	0.060	0.066	0.019	0.048	0.075	0.153	0.070	0.099	0.020	0.002	0.004	0.009	2.047	0.184	11.131	05550	
209	0.543	0.361	0.387	0.430	0.925	1.009	1.158	1.030	1.450	1.204	1.492	1.382	2.395	3.212	0.746	05540	
214	0.137	0.235	0.150	0.174	0.364	0.328	0.350	0.347	0.387	0.314	0.362	0.354	1.995	2.036	0.980	05540	
217	0.470	0.387	0.426	0.428	0.284	0.267	0.303	0.285	0.233	0.151	0.144	0.176	0.666	0.412	1.618		
218	0.024	0.039	0.028	0.030	0.117	0.094	0.133	0.115	0.174	0.142	0.140	0.152	3.807	5.039	0.755	11780	
219	0.029	0.056	0.060	0.048	0.103	0.132	0.103	0.112	0.142	0.139	0.125	0.135	2.320	2.797	0.830	11790	
221	0.116	0.029	0.103	0.083	0.245	0.168	0.209	0.207	0.097	0.081	0.076	0.084	2.505	1.020	2.455	10160	
222	0.683	0.702	0.746	0.710	0.411	0.278	0.364	0.351	0.154	0.168	0.141	0.155	0.495	0.218	2.274		
223	0.052	0.066	0.064	0.061	0.060	0.071	0.039	0.057	0.168	0.195	0.220	0.194	0.941	3.206	0.294		
228	0.017	0.011	0.017	0.015	0.215	0.211	0.214	0.213	0.057	0.060	0.030	0.049	14.384	3.291	4.371	08110	
230	0.144	0.072	0.159	0.125	0.193	0.193	0.147	0.178	0.275	0.292	0.371	0.312	1.420	2.498	0.569		
232	1.745	0.169	1.479	1.131	3.439	4.633	3.936	4.003	5.530	7.574	5.582	6.229	3.539	5.508	0.643	03000	
234	0.054	0.195	0.030	0.093	0.231	0.401	0.460	0.364	0.114	0.298	0.180	0.197	3.903	2.118	1.843		
236	0.898	0.301	0.791	0.664	0.466	0.428	0.577	0.490	0.117	0.202	0.182	0.167	0.739	0.252	2.931	05540	
238	0.029	0.016	0.045	0.030	0.107	0.118	0.098	0.108	0.047	0.019	0.029	0.032	3.581	1.062	3.371		
241	1.006	1.025	0.810	0.947	0.537	0.758	0.579	0.625	0.085	0.166	0.189	0.147	0.660	0.155	4.256	05540	
242	0.191	0.206	0.237	0.211	0.428	0.401	0.480	0.437	0.033	0.033	0.126	0.064	2.068	0.304	6.812		
189	0.154	0.266	0.178	0.199	0.033	0.097	0.079	0.070	0.032	0.014	0.052	0.033	0.349	0.163	2.138	05450	
