# Supplementary material for: The catabolism of 3,3’-thiodipropionic acid in Variovorax paradoxus strain TBEA6: A proteomic analysis
Source: PLoS One. 2019 Feb 11;14(2):e0211876. doi: 10.1371/journal.pone.0211876 (PMC6370202; doi:10.1371/journal.pone.0211876)
Supplement: S4 Table — Displayed are the normalized and mean normalized spot volumes of spots on gels from cultivations with gluconate in contrast to TDP and 3SP, ratios of mean normalized volumes, and detected proteins (MALDI-TOF-MS/MS) within respective spots (Accession number). Some accession numbers are missing for spots that were not identified via MALDI-TOF-MS/MS. Identified spots from the already described TDP metabolism are highlighted in blue; protein spots that belong to genes deleted during this study are highlighted in orange; proteins from both, which were found in the same spot, are marked in grey. (PDF) [file pone.0211876.s004.pdf]

S4 Table: Quantitation table of the second biological experiment. Displayed are the normalized and mean normalized spot volumes of spots on gels from cultivations with gluconate in contrast to TDP and 3SP, ratios of mean normalized volumes, and detected proteins (MALDI-TOF-MS/MS) within respective spots (Accession number). Some accession numbers are missing for spots that were not identified via MALDI-TOF-MS/MS. Identified spots from the already described TDP metabolism are highlighted in blue; protein spots that belong to genes deleted during this study are highlighted in orange; proteins from both, which were found in the same spot, are marked in grey.

Spot number	Normalized Volume Gluc (Vol%)			Mean Norm. Volume Gluc (Vol%)	Normalized Volume TDP (Vol%)			Mean Norm. Volume TDP (Vol%)	Normalized Volume 3SP (Vol%)			Mean Norm. Volume 3SP (Vol%)	Ratio of mean Norm. Volumes (Vol%/Vol%)			Acession numbers (VPARA_XXXXX)
													TDP / Gluc	3SP / Gluc	TDP / 3SP	
2	0,034	0,057	0,048	0,046	0,011	0,012	0,007	0,010	0,006	0,002	0,007	0,005	0,216	0,105	2,055	
4	0,082	0,195	0,094	0,124	0,128	0,120	0,129	0,126	0,913	0,889	0,857	0,887	1,015	7,160	0,142	66700
5	0,046	0,057	0,039	0,047	0,088	0,057	0,083	0,076	0,098	0,152	0,148	0,133	1,607	2,816	0,571	04900
7	0,011	0,037	0,037	0,028	0,045	0,039	0,047	0,044	0,074	0,076	0,081	0,077	1,569	2,748	0,571	68060
8	0,029	0,031	0,036	0,032	0,127	0,138	0,085	0,117	0,056	0,054	0,033	0,048	3,649	1,492	2,446	05770
9	0,041	0,091	0,092	0,075	0,097	0,119	0,082	0,099	0,182	0,200	0,171	0,184	1,330	2,464	0,540	07950
11	0,032	0,024	0,045	0,034	0,062	0,076	0,074	0,071	0,002	0,011	0,026	0,013	2,085	0,385	5,415	07950
12	0,173	0,211	0,206	0,197	0,780	0,790	0,860	0,810	0,943	0,810	1,069	0,941	4,120	4,786	0,861	21730
13	0,066	0,053	0,128	0,082	0,143	0,143	0,142	0,143	0,024	0,058	0,035	0,039	1,733	0,477	3,637	
14	0,036	0,025	0,035	0,032	0,027	0,020	0,043	0,030	0,061	0,061	0,081	0,068	0,943	2,105	0,448	42410 46440
15	0,018	0,032	0,063	0,038	0,096	0,105	0,108	0,103	0,103	0,102	0,129	0,111	2,727	2,950	0,924	04890
16	0,028	0,028	0,024	0,026	0,067	0,042	0,045	0,051	0,001	0,009	0,011	0,007	1,947	0,261	7,455	
17	0,051	0,031	0,044	0,042	0,020	0,052	0,020	0,031	0,072	0,101	0,108	0,094	0,729	2,215	0,329	05530
18	0,088	0,062	0,057	0,069	0,229	0,174	0,161	0,188	0,010	0,008	0,022	0,013	2,721	0,194	14,055	05580

19	0,061	0,076	0,095	0,077	1,398	1,470	1,410	1,426	1,637	1,403	1,639	1,560	18,436	20,164	0,914	03000		
20	0,013	0,013	0,016	0,014	0,043	0,036	0,025	0,034	0,000	0,010	0,016	0,009	2,446	0,608	4,024			
21	0,122	0,150	0,123	0,132	0,545	0,582	0,500	0,542	0,375	0,356	0,376	0,369	4,117	2,801	1,470	03010		
23	0,073	0,081	0,032	0,062	0,202	0,150	0,211	0,188	0,003	0,004	0,017	0,008	3,028	0,129	23,412			
24	0,015	0,031	0,038	0,028	0,157	0,178	0,160	0,165	0,145	0,129	0,153	0,142	5,889	5,080	1,159	03010		
26	0,090	0,113	0,079	0,094	0,154	0,156	0,272	0,194	0,032	0,034	0,023	0,030	2,068	0,317	6,528	12060		
28	0,044	0,063	0,054	0,054	0,098	0,112	0,113	0,108	0,096	0,093	0,106	0,098	2,003	1,831	1,093			
29	0,027	0,026	0,028	0,027	0,070	0,069	0,089	0,076	0,026	0,032	0,029	0,029	2,829	1,094	2,585	17940		
30	0,008	0,021	0,073	0,034	0,883	0,888	0,876	0,882	0,198	0,250	0,366	0,271	26,105	8,024	3,253	05600	05450	05540
31	0,060	0,057	0,028	0,049	0,176	0,211	0,163	0,183	0,046	0,036	0,023	0,035	3,773	0,719	5,250			
32	0,049	0,072	0,345	0,156	0,558	0,576	0,517	0,550	2,344	1,970	1,817	2,044	3,537	13,133	0,269	05490		
33	0,032	0,047	0,042	0,041	0,050	0,038	0,057	0,048	0,129	0,140	0,117	0,129	1,194	3,174	0,376			
36	0,064	0,030	0,035	0,043	0,024	0,038	0,018	0,027	0,177	0,201	0,180	0,186	0,615	4,308	0,143			
43	0,239	0,340	0,368	0,316	0,487	0,567	0,341	0,465	0,804	0,689	0,850	0,781	1,472	2,473	0,595			
47	0,064	0,055	0,047	0,056	0,047	0,117	0,113	0,092	0,297	0,308	0,162	0,256	1,663	4,604	0,361	05520		
48	0,096	0,153	0,137	0,128	0,704	0,748	0,637	0,696	0,525	0,545	0,871	0,647	5,419	5,038	1,076	04890		
50	0,082	0,108	0,061	0,083	0,022	0,022	0,053	0,032	0,013	0,012	0,007	0,011	0,387	0,129	2,990			
52	0,048	0,082	0,089	0,073	0,114	0,097	0,109	0,107	0,151	0,136	0,158	0,148	1,460	2,029	0,719	03280		
53	0,076	0,103	0,162	0,114	0,663	0,584	0,656	0,634	0,318	0,428	0,411	0,386	5,578	3,392	1,644	05440		
54	0,023	0,034	0,038	0,031	0,032	0,046	0,071	0,050	0,171	0,164	0,144	0,160	1,586	5,102	0,311	03190		
55	0,013	0,012	0,009	0,011	0,040	0,049	0,049	0,046	0,006	0,008	0,006	0,007	4,010	0,582	6,895	00950		
56	0,005	0,012	0,014	0,010	0,088	0,093	0,105	0,095	0,194	0,297	0,135	0,209	9,472	20,689	0,458	05540		
57	0,097	0,088	0,074	0,086	0,154	0,123	0,151	0,143	0,636	0,585	0,512	0,578	1,654	6,694	0,247	05770		
58	0,054	0,044	0,026	0,041	0,036	0,040	0,054	0,043	0,090	0,116	0,098	0,101	1,048	2,444	0,429	05600		
59	0,058	0,075	0,057	0,063	0,092	0,131	0,097	0,107	0,607	0,368	0,390	0,455	1,684	7,194	0,234	05540	05710	38090
60	0,042	0,044	0,034	0,040	0,116	0,123	0,116	0,119	0,082	0,071	0,101	0,085	2,986	2,129	1,403	68060		
61	0,024	0,031	0,019	0,025	0,042	0,038	0,044	0,041	0,057	0,068	0,056	0,060	1,680	2,447	0,687			
62	0,054	0,090	0,051	0,065	0,060	0,067	0,095	0,074	0,165	0,161	0,182	0,169	1,136	2,604	0,436	07950		
63	0,079	0,147	0,108	0,111	0,560	0,662	0,794	0,672	0,532	0,960	0,587	0,693	6,032	6,220	0,970	05540		

64	0,118	0,157	0,119	0,132	0,271	0,247	0,270	0,263	0,233	0,344	0,272	0,283	1,995	2,152	0,927	05770	
65	0,043	0,080	0,050	0,057	0,273	0,248	0,258	0,260	0,180	0,281	0,305	0,255	4,518	4,439	1,018	05540	
66	0,173	0,367	0,289	0,277	1,882	1,695	1,576	1,718	1,382	1,607	1,276	1,422	6,210	5,140	1,208	05540	
67	0,029	0,073	0,029	0,044	0,089	0,114	0,121	0,108	0,338	0,351	0,363	0,351	2,471	8,015	0,308	05690	
68	0,006	0,028	0,008	0,014	0,063	0,065	0,086	0,072	0,097	0,097	0,107	0,100	5,126	7,178	0,714	05540	05690
69	0,038	0,056	0,026	0,040	0,069	0,094	0,081	0,081	0,108	0,095	0,122	0,108	2,042	2,716	0,752		
72	0,052	0,141	0,166	0,120	0,999	1,039	0,952	0,997	1,221	1,126	1,073	1,140	8,307	9,500	0,874	04890	
75	0,032	0,054	0,042	0,043	0,109	0,110	0,109	0,109	0,131	0,129	0,150	0,136	2,557	3,193	0,801		
76	0,021	0,035	0,046	0,034	0,073	0,066	0,067	0,069	0,110	0,104	0,117	0,111	2,032	3,277	0,620		
77	0,003	0,011	0,010	0,008	0,021	0,024	0,021	0,022	0,027	0,025	0,027	0,026	2,785	3,339	0,834		
79	0,018	0,047	0,037	0,034	0,028	0,027	0,029	0,028	0,083	0,084	0,093	0,087	0,825	2,555	0,323	05510	
80	0,007	0,016	0,014	0,012	0,030	0,040	0,030	0,033	0,006	0,015	0,012	0,011	2,699	0,904	2,984	27770	
82	0,055	0,068	0,038	0,054	0,097	0,068	0,051	0,072	0,251	0,251	0,282	0,261	1,335	4,839	0,276	05550	04900
83	0,617	0,852	0,387	0,619	1,228	1,209	1,292	1,243	0,819	1,000	0,821	0,880	2,009	1,422	1,413	03010	
84	0,028	0,017	0,015	0,020	0,040	0,050	0,027	0,039	0,067	0,065	0,076	0,069	1,954	3,495	0,559	05520	
85	0,043	0,054	0,022	0,040	0,106	0,111	0,079	0,099	0,026	0,034	0,044	0,035	2,494	0,877	2,845	05550	
86	0,027	0,040	0,026	0,031	0,085	0,076	0,060	0,074	0,024	0,023	0,028	0,025	2,363	0,799	2,957	05550	
87	0,068	0,048	0,002	0,039	0,118	0,127	0,112	0,119	0,059	0,039	0,054	0,051	3,024	1,295	2,336	05550	
88	0,124	0,070	0,041	0,078	0,242	0,288	0,263	0,264	0,082	0,088	0,110	0,093	3,379	1,187	2,846	03280	05550
89	0,062	0,056	0,051	0,056	0,047	0,042	0,041	0,043	0,021	0,030	0,014	0,022	0,770	0,384	2,008		
100	0,088	0,120	0,150	0,120	0,038	0,045	0,057	0,047	0,015	0,017	0,029	0,020	0,390	0,168	2,318		
103	0,151	0,120	0,159	0,143	0,039	0,108	0,052	0,066	0,039	0,024	0,025	0,029	0,464	0,204	2,276	26530	10720
104	0,031	0,028	0,023	0,027	0,029	0,026	0,026	0,027	0,002	0,012	0,014	0,009	0,981	0,339	2,896	30850	
105	0,071	0,108	0,096	0,091	0,847	0,812	0,667	0,776	0,512	0,719	0,958	0,730	8,494	7,991	1,063	27730	
106	0,037	0,093	0,053	0,061	0,230	0,260	0,194	0,228	0,164	0,206	0,198	0,189	3,731	3,099	1,204	05550	
109	0,081	0,081	0,060	0,074	0,625	0,603	0,580	0,603	0,480	0,657	0,623	0,586	8,138	7,916	1,028	13010	
110	0,013	0,020	0,018	0,017	0,049	0,048	0,056	0,051	0,021	0,021	0,027	0,023	2,973	1,327	2,240	05710	
111	0,113	0,131	0,138	0,128	0,760	0,636	0,406	0,601	0,597	0,590	0,608	0,598	4,703	4,685	1,004	05710	
112	0,030	0,024	0,033	0,029	0,150	0,195	0,158	0,168	0,135	0,177	0,157	0,156	5,742	5,355	1,072	24900	27710

113	0,051	0,055	0,035	0,047	0,190	0,180	0,138	0,169	0,170	0,157	0,216	0,181	3,611	3,865	0,934	27760	61040
114	0,032	0,026	0,031	0,029	0,032	0,028	0,041	0,033	0,062	0,092	0,078	0,078	1,131	2,630	0,430	05490	05540
115	0,037	0,054	0,047	0,046	0,098	0,106	0,083	0,095	0,062	0,059	0,075	0,065	2,073	1,420	1,459	05700	10720
116	0,030	0,036	0,032	0,033	0,033	0,039	0,035	0,035	0,017	0,013	0,016	0,015	1,085	0,466	2,325		
117	0,044	0,033	0,017	0,032	0,096	0,109	0,107	0,104	0,021	0,027	0,037	0,029	3,292	0,904	3,643	11100	
118	0,018	0,031	0,034	0,028	0,069	0,067	0,085	0,074	0,040	0,031	0,038	0,036	2,679	1,319	2,030	03280	
119	0,034	0,039	0,016	0,030	0,087	0,066	0,077	0,077	0,038	0,030	0,033	0,034	2,573	1,132	2,274	03280	
120	0,127	0,093	0,093	0,104	0,256	0,248	0,320	0,275	0,092	0,139	0,129	0,120	2,638	1,151	2,291	03280	
121	0,029	0,042	0,053	0,041	0,245	0,188	0,127	0,186	0,113	0,126	0,159	0,133	4,527	3,220	1,406	27740	
123	0,020	0,022	0,017	0,020	0,062	0,054	0,046	0,054	0,009	0,019	0,022	0,017	2,751	0,854	3,221	05550	
124	0,020	0,046	0,023	0,030	0,069	0,061	0,078	0,069	0,034	0,025	0,019	0,026	2,302	0,864	2,664	26530	
128	0,346	0,250	0,183	0,260	0,619	0,567	0,668	0,618	0,055	0,095	0,082	0,077	2,380	0,298	7,991	33170	
131	0,117	0,123	0,090	0,110	0,417	0,385	0,457	0,420	0,449	0,517	0,446	0,471	3,813	4,278	0,891	04900	
132	0,055	0,041	0,026	0,040	0,054	0,048	0,029	0,043	0,091	0,118	0,098	0,102	1,074	2,531	0,425	05450	
138	0,038	0,064	0,046	0,050	0,209	0,195	0,276	0,226	0,087	0,110	0,115	0,104	4,564	2,094	2,180	05520	
149	0,023	0,034	0,022	0,026	0,075	0,050	0,052	0,059	0,003	0,004	0,006	0,005	2,268	0,175	12,949	05770	
150	0,046	0,043	0,030	0,040	0,029	0,030	0,024	0,028	0,010	0,009	0,011	0,010	0,699	0,248	2,823		
151	0,025	0,038	0,005	0,023	0,094	0,062	0,107	0,088	0,031	0,023	0,045	0,033	3,890	1,462	2,660	03190	
153	0,097	0,088	0,067	0,084	0,040	0,032	0,040	0,037	0,018	0,019	0,016	0,018	0,447	0,213	2,096	05600	05700
154	0,009	0,017	0,022	0,016	0,022	0,030	0,027	0,026	0,049	0,097	0,103	0,083	1,669	5,282	0,316	03190	
161	0,309	0,254	0,231	0,265	0,436	0,523	0,282	0,414	0,114	0,125	0,103	0,114	1,563	0,430	3,630	05440	
163	0,015	0,014	0,008	0,012	0,034	0,058	0,049	0,047	0,002	0,000	0,013	0,005	3,860	0,404	9,562	05550	
164	0,038	0,089	0,027	0,051	0,136	0,158	0,129	0,141	0,227	0,368	0,450	0,348	2,743	6,780	0,405	05550	
168	0,080	0,079	0,041	0,067	0,078	0,094	0,064	0,079	0,422	0,365	0,380	0,389	1,179	5,834	0,202	05490	
169	0,021	0,050	0,032	0,034	0,032	0,044	0,038	0,038	0,085	0,080	0,083	0,083	1,115	2,414	0,462	05690	
170	0,028	0,033	0,029	0,030	0,020	0,013	0,011	0,015	0,184	0,150	0,158	0,164	0,497	5,535	0,090	41350	
172	0,026	0,035	0,033	0,032	0,099	0,105	0,097	0,100	0,018	0,023	0,025	0,022	3,159	0,692	4,568	68060	
