# Supplementary material for: The catabolism of 3,3’-thiodipropionic acid in Variovorax paradoxus strain TBEA6: A proteomic analysis
Source: PLoS One. 2019 Feb 11;14(2):e0211876. doi: 10.1371/journal.pone.0211876 (PMC6370202; doi:10.1371/journal.pone.0211876)

S3 Fig.: **2D-gels from the second biological experiment with spot labels.** Cells cultivated with TDP and 3SP in comparison were disrupted and proteins extracted for 2D-gel analysis. Proteins (1.5 µg per gel) were firstly separated by isoelectric point (pH 5 to pH 8) and secondly by molecular weight. Proteins were stained with Coomassie Brilliant Blue, scanned, labeled, and analyzed via the Delta2D Software. Spot labels are displayed on the fused images.

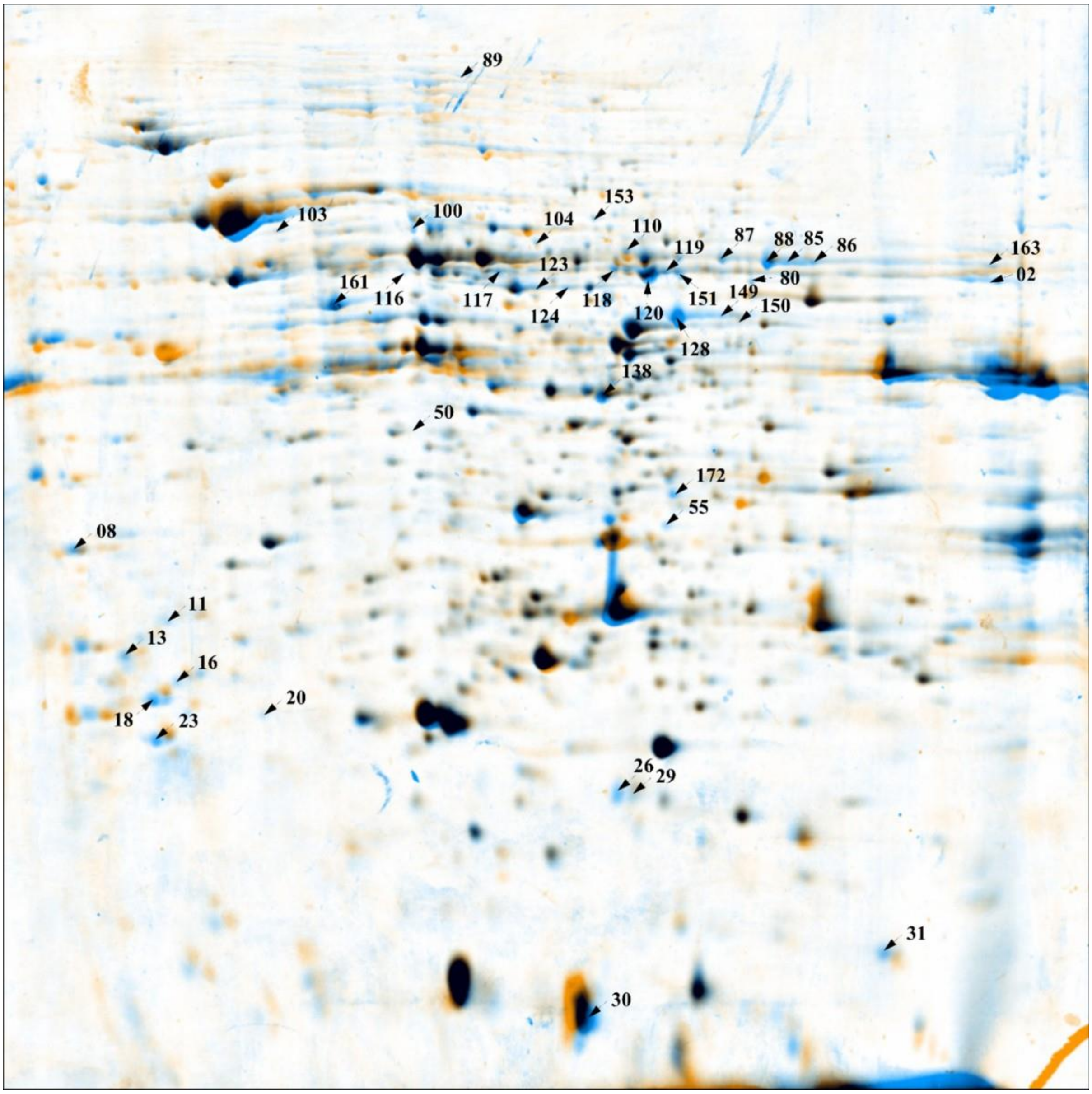

Supplement: S3 Fig — Cells cultivated with TDP and 3SP in comparison were disrupted and proteins extracted for 2D-gel analysis. Proteins (1.5 μg per gel) were firstly separated by isoelectric point (pH 5 to pH 8) and secondly by molecular weight. Proteins were stained with Coomassie Brilliant Blue, scanned, labeled, and analyzed via the Delta2D Software. Spot labels are displayed on the fused images. (PDF) [file pone.0211876.s007.pdf]
