# Supplementary material for: The catabolism of 3,3’-thiodipropionic acid in Variovorax paradoxus strain TBEA6: A proteomic analysis
Source: PLoS One. 2019 Feb 11;14(2):e0211876. doi: 10.1371/journal.pone.0211876 (PMC6370202; doi:10.1371/journal.pone.0211876)

S4 Fig.: Purification of Ech-30. Heterologous expression was performed using cells of *E. coli* BL21 (DE3) pLysS which were transformed with pET23a::*ech*-30. ZYP autoinduction medium was inoculated with freshly transformed cells and the cultures were incubated overnight at 30°C on a rotary shaker at 130 rpm. Purification was achieved using His Spin Trap columns (GE Healthcare) using 100 mM Tris/HCl buffer (pH 8.0 with 500 mM NaCl and different concentrations of imidazole. Cells were mixed with binding buffer (20 mM imidazole) before cell disruption and afterwards cell debris was removed by centrifugation (cell-free lysate). The cell-free lysate was loaded onto the columns (flow through) and washed with the above mentioned Tris/HCl Buffer with the following concentrations of imidazole: wash fraction I, 50 mM imidazole; wash fraction II, 100 mM imidazole; wash fraction III and IV, 200 mM imidazole. The protein was eluted using the Tris/HCl buffer with 500 mM imidazole.

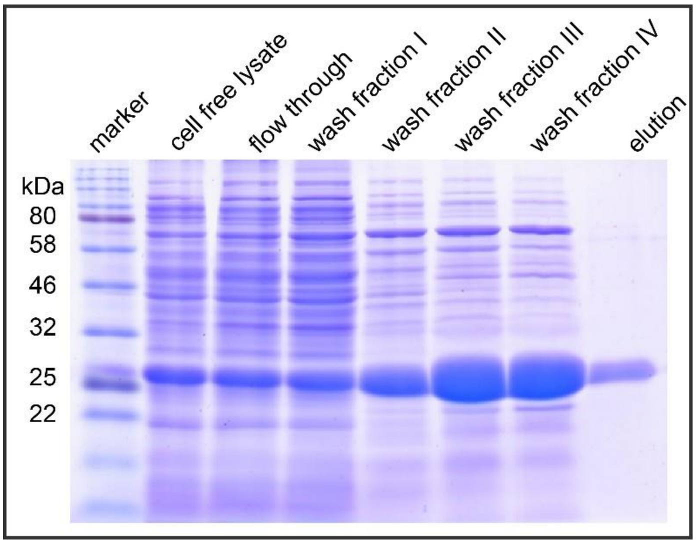

Supplement: S4 Fig — Heterologous expression was performed using cells of E. coli BL21 (DE3) pLysS which were transformed with pET23a::ech-30. ZYP autoinduction medium was inoculated with freshly transformed cells and the cultures were incubated overnight at 30°C on a rotary shaker at 130 rpm. Purification was achieved using His Spin Trap columns (GE Healthcare) using 100 mM Tris/HCl buffer (pH 8.0 with 500 mM NaCl and different concentrations of imidazole. Cells were mixed with binding buffer (20 mM imidazole) before cell disruption and afterwards cell debris was removed by centrifugation (cell-free lysate). The cell-free lysate was loaded onto the columns (flow through) and washed with the above mentioned Tris/HCl Buffer with the following concentrations of imidazole: wash fraction I, 50 mM imidazole; wash fraction II, 100 mM imidazole; wash fraction III and IV, 200 mM imidazole. The protein was eluted using the Tris/HCl buffer with 500 mM imidazole. (PDF) [file pone.0211876.s008.pdf]
