# Supplementary material for: The catabolism of 3,3’-thiodipropionic acid in Variovorax paradoxus strain TBEA6: A proteomic analysis
Source: PLoS One. 2019 Feb 11;14(2):e0211876. doi: 10.1371/journal.pone.0211876 (PMC6370202; doi:10.1371/journal.pone.0211876)

S5 Fig.: Thermal shift assay of purified Ech-30 using TDP (green) and TDP-CoA (red) as ligands. The blue curve represents the no ligand control. The lines without the peaks represent the no protein controls, also with addition of TDP (green) and TDP-CoA (red). The assay was performed in triplicate with 2 µM protein in presence of 2 mM TDP-CoA in 100 mM Tris/HCl (pH 8).

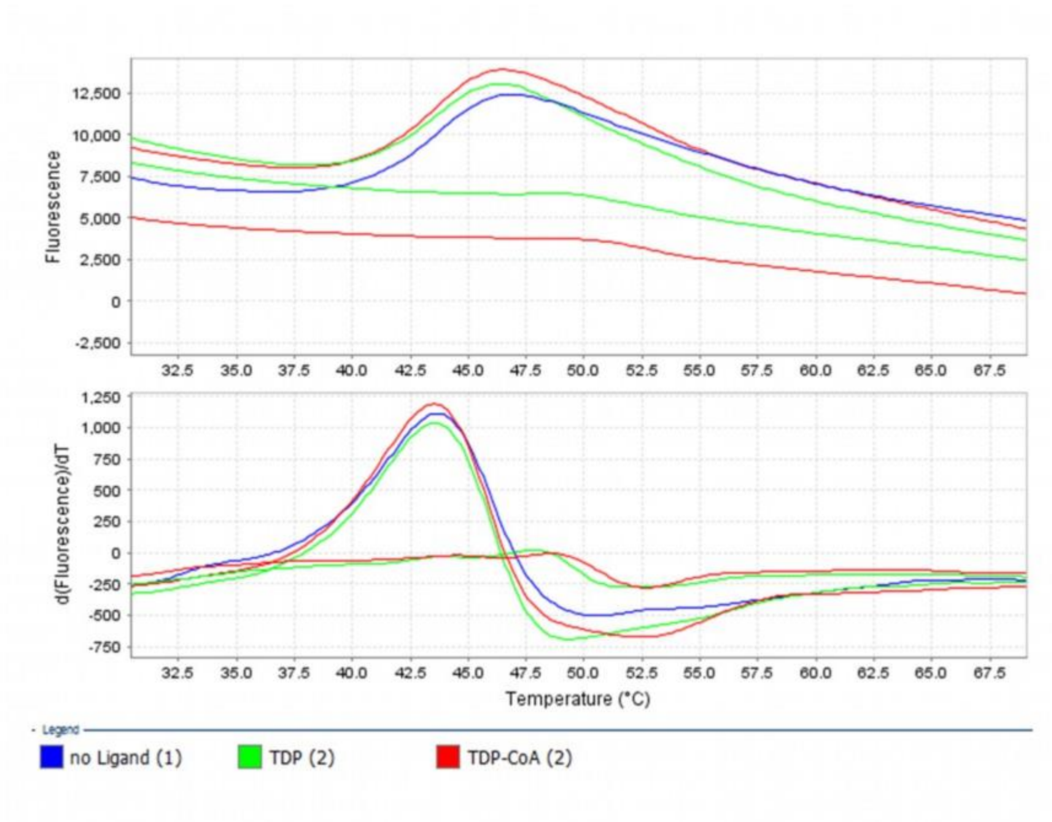

Supplement: S5 Fig — The blue curve represents the no ligand control. The lines without the peaks represent the no protein controls, also with addition of TDP (green) and TDP-CoA (red). The assay was performed in triplicate with 2 μM protein in presence of 2 mM TDP-CoA in 100 mM Tris/HCl (pH 8). (PDF) [file pone.0211876.s009.pdf]
